# Integrative bulk and single-cell transcriptomics links amino acid metabolism-related biomarkers to endothelial immune remodeling in periodontitis

**DOI:** 10.3389/fimmu.2026.1991235

**Published:** 2026-10-28

**Authors:** Haiwen Wu, Xuenan Liu, Yuan Zhu, Mudi Guo, Meng Wang, Meihua Chen, Xuejiao Liu

**Affiliations:** 1 Department of Periodontology, Shanghai Key Laboratory of Craniomaxillofacial Development and Diseases, Shanghai Stomatological Hospital & School of Stomatology, Fudan University, Shanghai, China; 2 Department of Prosthodontics, School and Hospital of Stomatology, Peking University, Beijing, China; 3 Department of Stomatology, Peking University Third Hospital, Beijing, China

**Keywords:** amino acid metabolism, diagnostic biomarkers, endothelial cells, immune infiltration, nomogram, periodontitis, single-cell RNA sequencing

## Abstract

**Background:**

Periodontitis (PD) is a chronic inflammatory disease driven by microbial dysbiosis and immune-mediated periodontal tissue destruction. Although amino acid metabolic dysregulation has been implicated in inflammatory diseases, its diagnostic relevance and cellular context in PD remain unclear. We aimed to identify amino acid metabolism-related diagnostic biomarkers and characterize their potential roles in PD.

**Methods:**

Bulk transcriptomic datasets (GSE16134, GSE10334, and GSE223924) and a single-cell RNA sequencing dataset (GSE164241) were analyzed. Amino acid metabolism-related differentially expressed genes were identified and functionally annotated through enrichment analyses. LASSO regression, Boruta, and random forest jointly screened diagnostic biomarkers. A nomogram was constructed and assessed by ROC, calibration, and decision curve analyses. Immune infiltration, consensus clustering, and weighted gene co-expression network analysis were performed; cell-type localization, cell-cell communication, and pseudotime analyses used single-cell data. Human gingival tissue samples were used for validation through histological analysis, immunohistochemistry, and quantitative real-time PCR (RT-qPCR).

**Results:**

Six amino acid metabolism-related biomarkers showed concordant expression changes across the three bulk datasets. TDO2, ICAM2, VWF and FABP4 were upregulated in periodontitis, whereas ACAT2 and AHNAK2 were downregulated. The six-gene nomogram discriminated PD from controls in GSE16134 (AUC = 0.956), while cross-platform evaluation revealed calibration limitations. Enrichment analysis linked these biomarkers to chemokine and cytokine signaling, cell adhesion and leukocyte transendothelial migration. Immune infiltration and consensus clustering revealed broad immune associations and three expression-based subgroups with distinct inflammatory features. Single-cell analysis localized the highest biomarker activity to endothelial cells, which showed the strongest diagnostic biomarker module score despite a relative reduction in their proportion. Pseudotime analysis revealed biomarker expression variation along an inferred endothelial trajectory, while CellChat predicted altered endothelial–immune communication. Experimental validation confirmed these changes for TDO2, ICAM2, VWF, FABP4 and ACAT2 at both mRNA and protein levels in clinical samples.

**Conclusion:**

We identify six amino acid metabolism-related candidate biomarkers with diagnostic potential for PD—five supported by clinical gingival tissue expression data—and link them to endothelial cell state changes and immune microenvironment remodeling. These findings provide an immune-metabolic framework for biomarker development and mechanistic studies in PD.

## 1 Introduction

PD ranks among the chronic non-communicable diseases and is driven by the interplay of periodontal microbial dysbiosis with host immune-inflammatory responses. It progressively destroys tooth-supporting tissues, including the gingiva, the periodontal ligament and the alveolar bone, and remains one of the major causes of adult tooth loss (1). In 2021, the Global Burden of Disease study put severe periodontitis at some 1.07 billion individuals, an age-standardized prevalence of about 12.5% (2). These data underscore the continuing global burden of PD. Mechanistically, PD is no longer viewed solely as a bacterial infection. It arises from interdependent processes involving microbial ecological imbalance, immune dysfunction, oxidative stress, and disrupted bone remodeling (3). Within periodontal lesions, epithelial cells, stromal cells, immune cells, and osteogenic lineage cells form a complex inflammatory regulatory network. Persistent activation of this network promotes pro-inflammatory mediator release and osteoclastogenesis downstream of receptor activator of nuclear factor kappa-B ligand (RANKL), so that bone formation becomes uncoupled from resorption (4).

Amino acid metabolism sits at the interface between nutrient availability and immune-inflammatory regulation (5). Beyond serving as substrates and nitrogen donors for protein synthesis, amino acids and their metabolites shape immune cell activation, macrophage polarization, T-cell differentiation and cytokine release through nutrient sensing, redox homeostasis, epigenetic regulation and receptor-dependent signaling (6, 7). In PD, metabolomic profiling of gingival tissue and gingival crevicular fluid has revealed broad amino acid abnormalities—involving citrulline, branched-chain amino acids and glutathione-related metabolites—several of which track with periodontal pocket depth and local inflammatory burden (8–11). These alterations are not host-intrinsic alone. The periodontal pocket, rich in inflammatory exudate, blood-derived components and tissue degradation products, sustains proteolytic anaerobes that catabolize amino acids into indole, biogenic amines, ammonia, hydrogen sulfide and methanethiol, products that in turn affect biofilm stability, epithelial barrier integrity and host inflammatory responses (12). The tryptophan-indole pathway of Porphyromonas gingivalis enhances bacterial virulence and aggravates gingival inflammation and alveolar bone resorption in mice (13), while elevated branched-chain amino acids induce MMP-2 and MMP-9 secretion in human periodontal ligament stem cells via NF-κB signaling (14). Amino acid metabolism therefore constitutes a shared axis between microbial pressure and host response. Yet existing work has largely examined individual metabolites or single pathogens, leaving the global expression, diagnostic relevance, immune associations and cell type-specific distribution of host amino acid metabolism-related genes (AAM-RGs) undefined.

Single-cell RNA sequencing (scRNA-seq) provides a strategy to address this gap at cell-level resolution. Periodontal tissue contains epithelial cells, fibroblasts, endothelial cells, immune cells and bone-associated cells, populations that differ in nutrient uptake, metabolic regulation, inflammatory signaling and tissue-remodeling functions. Endothelial cells occupy a particularly informative position in this context: they control the delivery of amino acids and other nutrients into inflamed tissue, and they govern leukocyte adhesion and recruitment, placing them directly at the junction of local metabolic supply and immune infiltration. Recent single-cell and spatial transcriptomic studies have shown that sulcular and junctional epithelial keratinocytes in PD lesions acquire abnormal differentiation and pro-inflammatory states, accompanied by immune-cell aggregation, enhanced fibroblast-immune interactions, and remodeled intercellular communication networks (15, 16). Bulk transcriptomic data, however, represent averaged signals from mixed tissues and cannot readily distinguish altered cell composition from intrinsic transcriptional regulation within specific cell types. Integrating bulk transcriptomics with scRNA-seq can therefore identify tissue-level amino acid metabolism-related expression signatures and localize their major cellular sources, clarifying how these signatures relate to the local immune microenvironment and periodontal tissue remodeling (17).

To systematically characterize host amino acid metabolism-related genes in PD, we brought bulk transcriptomic cohorts together with scRNA-seq data. We drew together differential expression testing, machine learning-based feature selection, then pathway enrichment, immune microenvironment profiling and single-cell annotation. Firstly, we identified PD-associated amino acid metabolism-related expression patterns at the tissue level and screened candidate diagnostic biomarkers. Next, we evaluated their associated functional pathways and immune microenvironmental features. Finally, we localized the major cell types expressing key genes and explored their potential cell-cell communication patterns. This study provides an immune-metabolic framework for understanding amino acid metabolic dysregulation in PD. It also identifies candidate targets for future mechanistic validation and diagnostic biomarker evaluation.

## 2 Materials and methods

### 2.1 Data collection

All transcriptomic datasets were obtained from the NCBI Gene Expression Omnibus (GEO; https://www.ncbi.nlm.nih.gov/geo/). Two microarray datasets, GSE16134 and GSE10334, were generated on the GPL570 platform (Affymetrix Human Genome U133 Plus 2.0 Array), and one bulk RNA-sequencing dataset, GSE223924, on the GPL24676 platform (Illumina NovaSeq 6000). GSE16134 served as the training dataset, comprising 310 gingival tissue samples from 241 PD-affected and 69 clinically unaffected sites. GSE10334 (183 affected-site and 64 clinically unaffected-site samples) was used for supplementary reproducibility assessment because its source cohort partially overlapped with that of GSE16134. GSE223924 served as an independent validation dataset, including gingival tissue samples from patients with periodontitis and healthy controls; peri-implantitis samples were excluded. The reference samples in GSE16134 and GSE10334 were obtained from clinically unaffected gingival sites in patients with periodontitis, whereas GSE223924 used healthy gingival tissues as controls. For GSE223924, the 10 peri-implantitis samples were excluded and the remaining 20 samples (10 periodontitis, 10 healthy) retained. Samples were assigned to the periodontitis (PD) or control (CT) group according to the site-status annotations in the original GEO metadata. Owing to platform heterogeneity, the microarray and RNA-sequencing data were normalized separately rather than merged, with GSE223924 counts transformed to log2(TPM + 1).

We analyzed 13 healthy gingival samples from 13 donors (21–61 years; 10 females and 3 males) and 8 periodontitis gingival samples from 5 donors (34–45 years; 3 females and 2 males) from GSE164241. The eight healthy buccal mucosa samples were not included in this analysis. Donor information was obtained from the associated Human Cell Atlas metadata. The original study collected gingival collar biopsies from healthy individuals and patients with moderate-to-severe periodontitis. In the original study, healthy gingival biopsies were obtained from sites with probing depths <3 mm and no bleeding on probing. Its exclusion criteria included tobacco use within 1 year before screening and use of systemic antibiotics, systemic or inhaled corticosteroids, other immunosuppressants, chemotherapeutic agents, or high-dose commercial probiotics within 3 months before enrollment (37). Individual periodontal stage/grade, complete smoking and medication histories, and exact tooth-level sampling sites were not reported in the public sources examined.

The amino acid metabolism-related gene (AAM-RG) set was obtained from the curated gene list reported in reference (18). That study integrated GeneCards database entries with PubMed literature retrieval and identified 1,204 AAM-RGs (**Supplementary Table S1**). Gene symbols were standardized before downstream analysis. The standardized AAM-RGs were matched to genes detected in the GSE16134 expression matrix. For each sample, the AAM-RG score was calculated as the arithmetic mean expression of the 1,091 matched genes. The GSE16134 differentially expressed genes (DEGs) were overlaid on the AAM-RG inventory, and the overlap defined the amino acid metabolism-related differentially expressed genes (AAMRDGs). **Figure 1** outlines the workflow.

**Figure 1:**
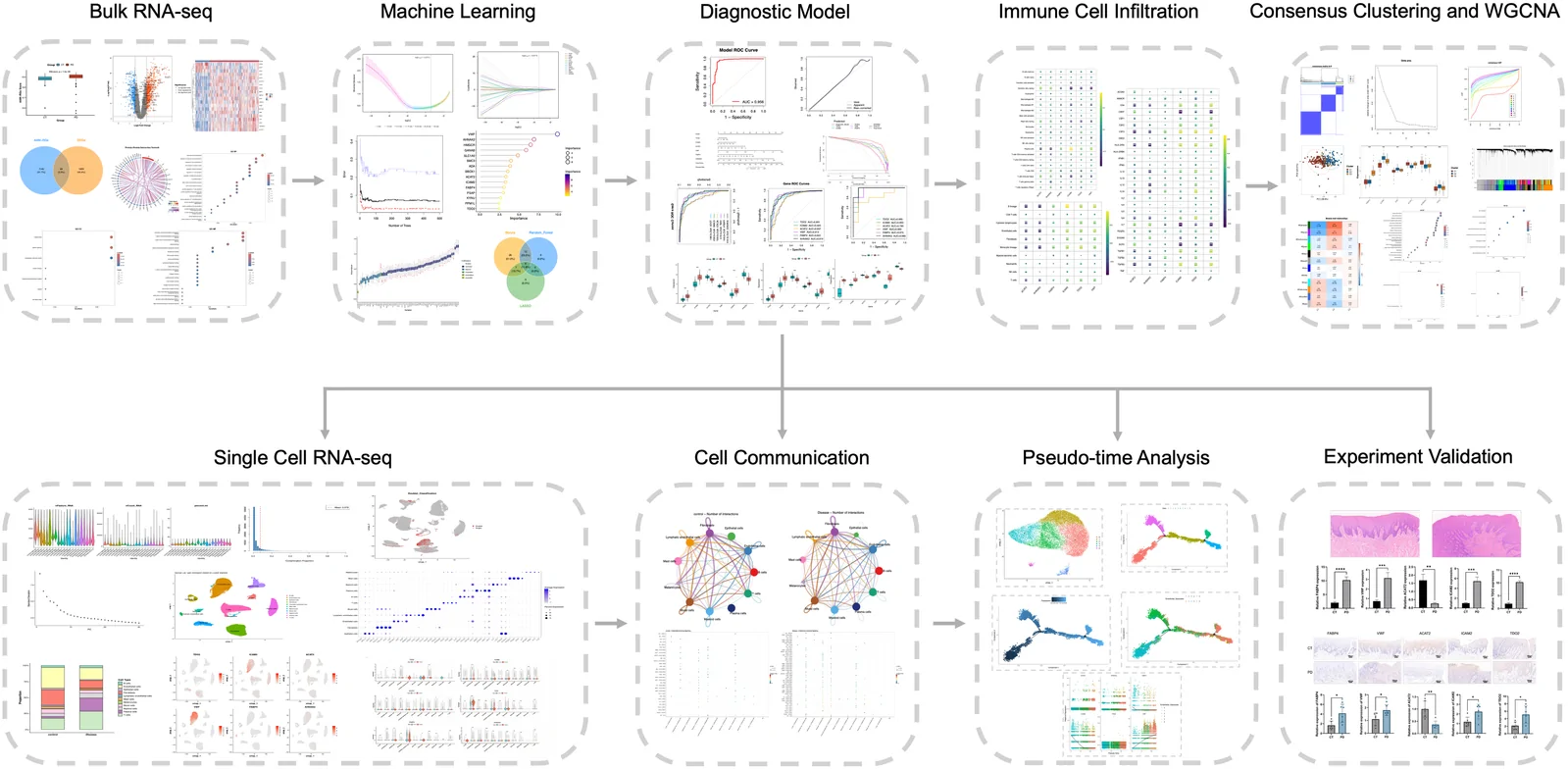
Overall analytical workflow of this study.

### 2.2 Identification of AAMRDGs

Samples in GSE16134 were assigned to the PD and CT groups according to the original GEO annotations. Differential expression between PD and CT samples of GSE16134 was tested with limma (v3.66.0) (19). Transcripts meeting both thresholds — |log2 fold change| > 0.5 and Benjamini–Hochberg adjusted P <0.05 — were taken as differentially expressed genes (DEGs) and rendered as a volcano plot with ggplot2 v4.0.1 (20). DEGs were intersected with the AAM-RG set to obtain PD-associated AAMRDGs. The overlap was visualized using VennDiagram (v1.7.3) (21). Expression patterns of AAMRDGs in the PD and CT groups were visualized using pheatmap (v1.0.13) (22).

### 2.3 Functional enrichment and protein-protein interaction network analysis

The 64 AAMRDGs were annotated functionally in clusterProfiler v4.18.4 (23), with org.Hs.eg.db supplying the gene identifiers. Two ontologies were queried: Gene Ontology (GO), across its biological process (BP), cellular component (CC) and molecular function (MF) branches, and the Kyoto Encyclopedia of Genes and Genomes (KEGG). Benjamini–Hochberg correction was applied to all raw P values. Only terms below an adjusted P of 0.05 were reported.

Protein-protein interaction (PPI) data for the AAMRDGs came from STRING (https://string-db.org/). The search was restricted to Homo sapiens (taxonomy ID 9606). Edges scoring below a confidence of 0.4 were discarded. Isolated nodes were dropped as well, so that every gene displayed retained at least one edge (degree ≥ 1). circlize (v0.4.17) (24) then drew the network in R 4.5.2.

### 2.4 Machine learning-based identification of core candidate genes

The 64 AAMRDGs identified in the GSE16134 training dataset were used as input features for machine learning-based feature selection. Three algorithms were applied: least absolute shrinkage and selection operator (LASSO) regression, random forest (RF), and Boruta. A binomial LASSO model was constructed using glmnet (v4.1.10) (25), with α = 1. Predictor variables were standardized, and the penalty parameter was selected by 10-fold cross-validation using binomial deviance as the performance measure. Genes with nonzero coefficients at the one-standard-error penalty (λ_1se_ = 0.03434; log(λ_1se_) = −3.3715) were retained.

The RF model was constructed using randomForest (v4.7.1.2) (26), with mtry = 8, nodesize = 1, and bootstrap sampling with replacement. Five hundred trees were grown initially. The count was then reset to whichever value gave the lowest out-of-bag error, subject to a floor of 50. Importance was read off MeanDecreaseGini, and any gene scoring above 2 was kept by RF.

Boruta feature selection was performed using Boruta (v8.0.0) (27). The maximum number of iterations was set to 500, and 500 trees were used for the embedded random forest. Tentative features were resolved using TentativeRoughFix. Genes classified as Confirmed were defined as Boruta-selected genes. The overlapping genes selected by LASSO, RF, and Boruta were identified using VennDiagram (v1.7.3) and defined as core candidate genes.

### 2.5 Validation of diagnostic biomarker expression

Each core candidate was compared between PD and CT samples by Wilcoxon rank-sum test. The six core candidate genes identified in GSE16134 were assessed for differential expression (P < 0.05) and consistency of expression direction across GSE16134, GSE10334 and GSE223924.

### 2.6 Construction and evaluation of the diagnostic nomogram

To evaluate the combined diagnostic value of the diagnostic biomarkers, a multivariable logistic regression model was constructed in the GSE16134 training dataset. Biomarker expression levels were used as predictors, and disease status was used as the binary outcome. The model was fitted using the lrm function in the rms package (v8.1.0) (28).

A nomogram was generated from the fitted logistic regression model. Each biomarker was assigned a score according to its expression level and regression coefficient. Scores across biomarkers were summed to obtain a total score, which was then converted into the predicted probability of PD. A higher total score indicated a higher predicted probability of PD. A nomogram with variable distributions and individual prediction markers was also plotted using regplot (v1.1) (29).

For the model fitted in GSE16134, odds ratios (ORs) were calculated by exponentiating the regression coefficients, and multicollinearity was assessed using variance inflation factors (VIFs). The model was further evaluated in GSE10334 and GSE223924 with its regression coefficients and intercept held fixed. Discrimination was assessed by receiver operating characteristic (ROC) analysis using pROC (v1.19.0.1) (30), and areas under the curve (AUCs) were reported with 95% confidence intervals. Calibration was assessed using calibration curves, and decision curve analysis (DCA) was performed using rmda (v1.6) (31) to explore net benefit. Sensitivity and specificity were calculated using the cutoff determined in GSE16134, which was applied unchanged to GSE10334 and GSE223924.

### 2.7 Functional analysis of diagnostic biomarkers

To map functional relationships among the diagnostic biomarkers, a gene-gene interaction (GGI) network was retrieved from GeneMANIA (http://www.genemania.org/). The six biomarkers were entered as input genes, and GeneMANIA returned their links to functionally related genes under the human background.

Signaling pathways tied to each biomarker were sought by gene set enrichment analysis (GSEA) in the GSE16134 training data. The reference collection was the KEGG subset of the Molecular Signatures Database (MSigDB; https://www.gsea-msigdb.org/), human GMT release v2026.1. For a given biomarker, samples fell into a high- and a low-expression group at the median. limma (v3.66.0) then contrasted the two groups. The full gene list was ordered from the highest moderated t statistic downwards. clusterProfiler (v4.18.4) ran the enrichment on that ranked list. Benjamini–Hochberg correction was applied, with significance set at an adjusted P below 0.05. For each biomarker, the five pathways with the smallest adjusted P values were displayed as enrichment curves.

### 2.8 Immune infiltration and inflammatory gene correlation analysis

Two deconvolution tools, CIBERSORT (32) and MCP-counter (33), were applied to the GSE16134 gingival tissue profiles to characterize immune cell infiltration and the PD immune microenvironment. CIBERSORT ran under quantile normalization over 1,000 permutations, returning relative proportions for 22 immune cell subsets. MCP-counter instead yields abundance scores across 10 immune and stromal cell populations. Spearman correlations of the six biomarkers with inferred cell profiles and 28 predefined inflammation-related genes were assessed, with Benjamini–Hochberg correction applied separately to each correlation matrix (adjusted P < 0.05). Results were visualized as square bubble plots using ggplot2 (v4.0.1).

### 2.9 Identification of PD molecular subtypes

To investigate molecular heterogeneity among PD samples, consensus clustering was performed using the expression profiles of diagnostic biomarkers in PD samples from GSE16134. The analysis was conducted using ConsensusClusterPlus (v1.74.0) (34). Candidate cluster numbers were set from 2 to 11. For each clustering iteration, 80% of samples and all diagnostic biomarkers were resampled, and 50 resampling iterations were performed. Partitioning used k-means over Euclidean distance. The consensus matrix, the cumulative distribution function curve and the relative change in area under that curve together fixed the optimal cluster number. Subtype distribution was displayed by principal component analysis, and biomarker expression compared across subtypes by Kruskal–Wallis test. The resulting subtypes were regarded as expression-based subgroups for exploratory analyses within GSE16134.

### 2.10 Weighted gene co-expression network analysis and subtype-associated enrichment analysis

Weighted gene co-expression network analysis (WGCNA) served to define gene co-expression modules linked to PD molecular subtypes. The expression matrix of PD samples from GSE16134 was normalized, and only genes whose expression standard deviation exceeded 0.25 were retained. Outlier samples were assessed by hierarchical clustering. The pickSoftThreshold function picked a soft-thresholding power in the range 1–30 satisfying the scale-free topology criterion (fit index ≥ 0.85).

Unsigned adjacency and topological overlap matrices were then constructed. Co-expression modules came from average-linkage hierarchical clustering with dynamic tree cutting, at deepSplit 2 and a minimum module size of 50 genes. Similar modules were merged at a 0.25 threshold. Pearson correlation coefficients, with their P values, were computed between module eigengenes and PD molecular subtypes to identify subtype-associated modules.

GO BP, GO CC, GO MF and KEGG enrichment analyses were then run on subtype-associated module genes in clusterProfiler (v4.18.4). All genes included in the WGCNA analysis were used as the enrichment background. P values carried Benjamini–Hochberg adjustment, and an adjusted P < 0.05 was taken as statistically significant.

### 2.11 Processing and annotation of scRNA-seq data

The GSE164241 scRNA-seq samples included here were processed in Seurat (v5.3.0) (35). The 10X Genomics expression matrices were read for each sample, and cells were labeled as CT or PD according to sample names. Expression matrices from all included samples were merged to construct a Seurat object using CreateSeuratObject.

During quality control, genes detected in three or more cells were retained. Cells were likewise kept where they carried 200–4,000 detected genes with a mitochondrial gene percentage under 20%. Ambient RNA contamination was corrected using decontX, and cells with an estimated contamination fraction of 50% or higher were excluded. DoubletFinder was run separately on each sample, and only singlets were carried forward.

Normalization used NormalizeData under the LogNormalize method at a scale factor of 10,000. FindVariableFeatures, run with the vst method, returned 2,000 highly variable genes. ScaleData then centered and scaled the data ahead of principal component analysis. Harmony corrected batch effects across samples. In Harmony space, a nearest-neighbor graph was built from the first 20 dimensions. Multi-resolution clustering was performed across resolutions from 0.1 to 1.0. Based on clustree evaluation, the final clustering resolution was set to 0.1. Cell distributions were displayed by UMAP. Nineteen cells were excluded before final cell-type annotation and downstream analyses.

FindAllMarkers returned positive marker genes per cluster by Wilcoxon rank-sum test, at min.pct = 0.25 and logfc.threshold = 0.25. Adjusted P < 0.05 served as the screening criterion. Cell types were annotated by hand from highly expressed marker genes, canonical cell markers, and previous literature (36, 37). Annotation results and marker expression patterns were displayed using UMAP plots, bubble plots, and heatmaps. Cell-type composition in CT and PD samples was summarized descriptively at both sample and group levels.

### 2.12 Identification of key cell types

To identify the key cell types, cell-type composition in the CT and PD groups was first compared descriptively. The observed/expected ratio (Ro/e) of each cell type was then obtained as its observed cell count over the count expected from the group-by-cell-type contingency table, as described previously (38). Values above 1 indicated relative enrichment, whereas values below 1 indicated relative underrepresentation.

Within each annotated cell type, expression differences in diagnostic biomarkers between PD and CT cells were tested by Wilcoxon rank-sum test. Significance was set at P < 0.05. Cell types in which all diagnostic biomarkers showed between-group expression differences were defined as candidate key cell types.

Seurat’s AddModuleScore function supplied a diagnostic biomarker module score for every cell. Candidate cell types were ranked by integrating three criteria: the number and direction of significant biomarker differences, mean diagnostic biomarker ModuleScore, and biomarker expression activity in PD cells. The cell type with the strongest overall biomarker activity was designated the key cell type. Seurat DotPlot and ggplot2 displayed average expression levels and percentages of expressing cells for each diagnostic biomarker across cell types.

### 2.13 Cell-cell communication and pseudotime analyses

Cell-cell communication was analyzed on the cell-type-annotated scRNA-seq dataset. CellChat (v2.1.2) (39) drew on CellChatDB.human as the ligand-receptor reference. The standard CellChat pipeline was followed: subsetData, identification of overexpressed signaling genes and of overexpressed ligand-receptor pairs, computation of communication probabilities using the triMean method, filtering of communications, pathway-level probability calculation, network aggregation, and centrality analysis. Ligand-receptor interactions at P < 0.05 were taken as significant.

To compare cell-cell communication between PD and CT groups, CellChat objects were constructed separately for each group. Only cell types present in both groups and represented by at least 20 cells per group were retained. The PD and CT CellChat objects were merged to compare communication number, communication strength, and pathway information flow. Incoming and outgoing ligand-receptor relationships were further extracted when key cells served as signal receivers or senders.

To analyze transcriptional heterogeneity and pseudotime progression of the key cell type, the key cells were extracted and reprocessed. The key cell subset was normalized, scaled, batch-corrected with Harmony, reclustered across resolutions from 0.1 to 1.0, and visualized by UMAP. The final clustering resolution was set to 0.2 based on clustree evaluation. Positive marker genes for key cell subclusters were identified using FindAllMarkers with only.pos = TRUE and min.pct = 0.25.

Monocle 2 (v2.36.0) (40) was used to infer pseudotime trajectories for key cells. Dimensionality reduction was performed using DDRTree, with orig.ident included as a residual term to reduce sample-origin effects. Cells were ordered using orderCells without manually specifying a root state. Key cell subclusters, sample groups, diagnostic biomarker expression, and dynamic expression patterns along pseudotime were visualized.

### 2.14 Human periodontal tissue samples

A total of 40 gingival tissue specimens were collected at the Dental Hospital of Fudan University from a single clinical cohort comprising 20 patients with periodontitis and 20 healthy controls. RT-qPCR, H&E staining, and immunohistochemistry each included specimens from 10 distinct donors per group. Tissue specimens from some donors were used for both H&E staining and immunohistochemistry. All participants provided written informed consent. Ethical approval and study registration are detailed in the Ethics statement.

### 2.15 Histological analysis (H&E staining)

Gingival tissues were first fixed in 4% paraformaldehyde, then dehydrated and paraffin-embedded. Serial sections of 4–5 μm were then cut. For H&E staining, sections were cleared of paraffin in xylene, then rehydrated down a graded ethanol series. Nuclei were stained in Harris hematoxylin, differentiated with 1% acid-alcohol, and blued in an alkaline solution. Sections were counterstained in alcoholic eosin-Y, dehydrated, xylene-cleared and mounted under neutral medium. Histological features including epithelial structure, connective tissue collagen distribution and inflammatory cell infiltration were observed under a light microscope.

### 2.16 Immunohistochemistry

Tissue sections were first dewaxed in xylene, then rehydrated through graded alcohol. Sections were washed in phosphate-buffered saline (PBS), then held in 3% hydrogen peroxide for 10 min to quench endogenous peroxidase activity, followed by a further PBS wash. Goat serum blocked the sections for 20 min before overnight incubation at 4 °C with the primary antibodies: TDO2 (K112875P; Solarbio, Beijing, China), ICAM2 (2096; GenuIN Biotech, Hefei, China), ACAT2 (61849; GenuIN Biotech, Hefei, China), VWF (GB11020; Servicebio, Wuhan, China), FABP4 (ab102606; Aladdin, Shanghai, China). After three further washes, the sections received secondary antibody (PV-9003; Zhongshan, Beijing, China) together with avidin-biotin-peroxidase complexes for 10 min. The sections were then counterstained in hematoxylin, dehydrated and mounted onto glass slides. For negative controls, PBS replaced the primary antibodies on each sample. Similarly, isotype controls used goat IgG (B900630; Proteintech Group, Wuhan, China) on parallel slides.

### 2.17 RT-qPCR

Reverse transcription-quantitative real-time PCR (RT-qPCR) served to confirm diagnostic biomarker expression in clinical gingival tissue samples. Gingiva from 20 patients was dissected within 3 minutes on ice after isolation. Calculus, blood clots and bone fragments were removed and samples were cut into small pieces (1–3 mm). Twenty milligrams of gingiva was transferred into grinding tubes holding zirconia beads plus 1 ml TRIzol (Invitrogen, Carlsbad, CA, USA). Cryogenic homogenization conditions: 30 Hz, 40 s grinding and 90 s pause (heat dissipation), until no visible large tissue fragments. RNA concentration was read on a NanoDrop 8000 spectrophotometer (Thermo Fisher Scientific, Waltham, MA, USA) from A260, with the 260:280 ratio taken as the purity index. Total RNA then went into first-strand cDNA synthesis with the FastKing One Step RT-PCR Kit (TIANGEN BIOTECH, Beijing, China), used as the supplier specifies. Amplification ran on SuperReal PreMix Plus (TIANGEN BIOTECH, Beijing, China) under the same supplier protocol. Glyceraldehyde 3-phosphate dehydrogenase (GAPDH) served as the normalizer. Primer sequences for TDO2, ICAM2, ACAT2, VWF, FABP4 and GAPDH appear in **Supplementary Table S2**. Relative expression was derived by the 2^−ΔΔCt method.

### 2.18 Statistical analysis

Unless otherwise specified, all bioinformatics analyses ran in R 4.5.2. Two-group comparisons used the Wilcoxon rank-sum test except where another test is named for a particular analysis. Comparisons across more than two groups used the Kruskal–Wallis test. Correlations were assessed by Spearman or Pearson correlation, as specified in each analysis. P values from analyses involving multiple testing carried Benjamini–Hochberg adjustment. Statistical significance was set at P < 0.05, or at adjusted P < 0.05 where adjustment applied. R packages and their version numbers are given in the corresponding Methods sections. For RT-qPCR validation, Ct-derived relative expression values were analyzed in GraphPad Prism 5 by unpaired two-sided t test.

## 3 Results

### 3.1 Identification of 64 AAMRDGs

To assess amino acid metabolism-related transcriptional alterations in PD, AAM-RG scores were first compared between the PD and CT groups in GSE16134. AAM-RG scores were significantly higher in the PD group than in the CT group, indicating higher mean expression of the included AAM-RGs (**Figure 2A**). Differential expression analysis identified 1,067 DEGs using the thresholds |log2FC| > 0.5 and adjusted P < 0.05, of which 665 were upregulated and 402 downregulated (**Figure 2B**). Overlaying these DEGs on AAM-RGs yielded 64 AAMRDGs (**Supplementary Table S3**; **Figure 2C**). Heatmap analysis further showed distinct expression patterns for the top-ranked 20 AAMRDGs across PD and CT samples (**Figure 2D**).

**Figure 2:**
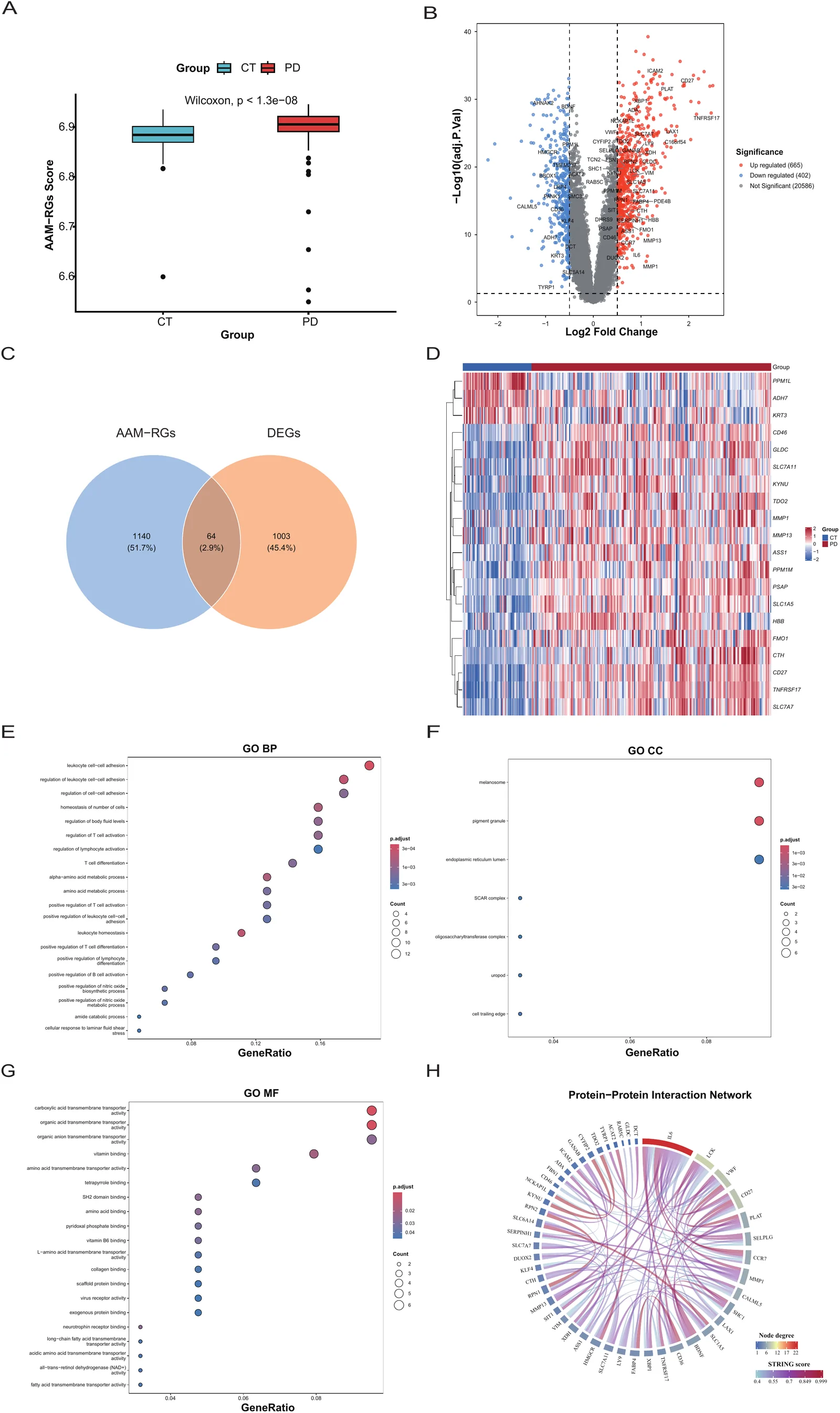
AAM-RGs screening and functional enrichment analysis. **(A)** Differences in AAM-RG scores between the PD group and the CT group. **(B)** Volcano plot of DEGs between the PD group and the CT group. Red dots, significantly upregulated genes; blue dots, significantly downregulated genes; gray dots, genes with no significant difference. **(C)** Intersection analysis of DEGs and AAM-RGs. **(D)** Heatmap of top20 of AAMRDGs in the datasets. **(E-G)** Gene Ontology (GO) functional enrichment analysis of AAMRDGs. **(H)** Construction of PPI network.

### 3.2 Enrichment and PPI network analysis of the 64 AAMRDGs

Annotation of the 64 AAMRDGs returned 231 significant GO terms at adjusted P < 0.05 — 203 biological process (BP), 21 molecular function (MF) and 7 cellular component (CC) terms. BP terms were concentrated in cell-cell adhesion between leukocytes, activation and differentiation of lymphocytes and T cells, leukocyte homeostasis, and amino acid and alpha-amino acid metabolic processes. CC terms included melanosomes, pigment granules, the endoplasmic reticulum lumen, and the oligosaccharyltransferase complex. MF terms were enriched for amino acid, organic acid, and carboxylic acid transmembrane transporter activity, as well as amino acid, vitamin B6, and pyridoxal phosphate binding (**Figures 2E–G**; **Supplementary Table S4**). No KEGG pathway reached significance under the same screening criteria.

The 64 AAMRDGs were then assembled into a STRING PPI network at a confidence of ≥ 0.4. IL6, LCK, CD27, and VWF had multiple protein-level interactions, and IL6 showed the highest degree in the network (**Figure 2H**). These findings linked PD-associated AAMRDGs primarily to immune-cell activation, cell adhesion, and amino acid transport-related processes.

### 3.3 Machine learning identified and validated six diagnostic biomarkers

The 64 AAMRDGs were next used for machine learning-based feature selection. LASSO regression retained 13 genes under the one-standard-error criterion (**Figures 3A, B**). Random forest analysis selected 18 genes according to variable importance scores. The out-of-bag error curve is shown in **Figure 3C**, and **Figure 3D** displays the 15 highest-ranked genes among the 18 RF-selected genes. Boruta analysis identified 51 confirmed genes (**Figure 3E**). Intersecting the genes selected by the three algorithms yielded six core candidate genes: TDO2, ICAM2, ACAT2, VWF, FABP4, and AHNAK2 (**Figure 3F**).

**Figure 3:**
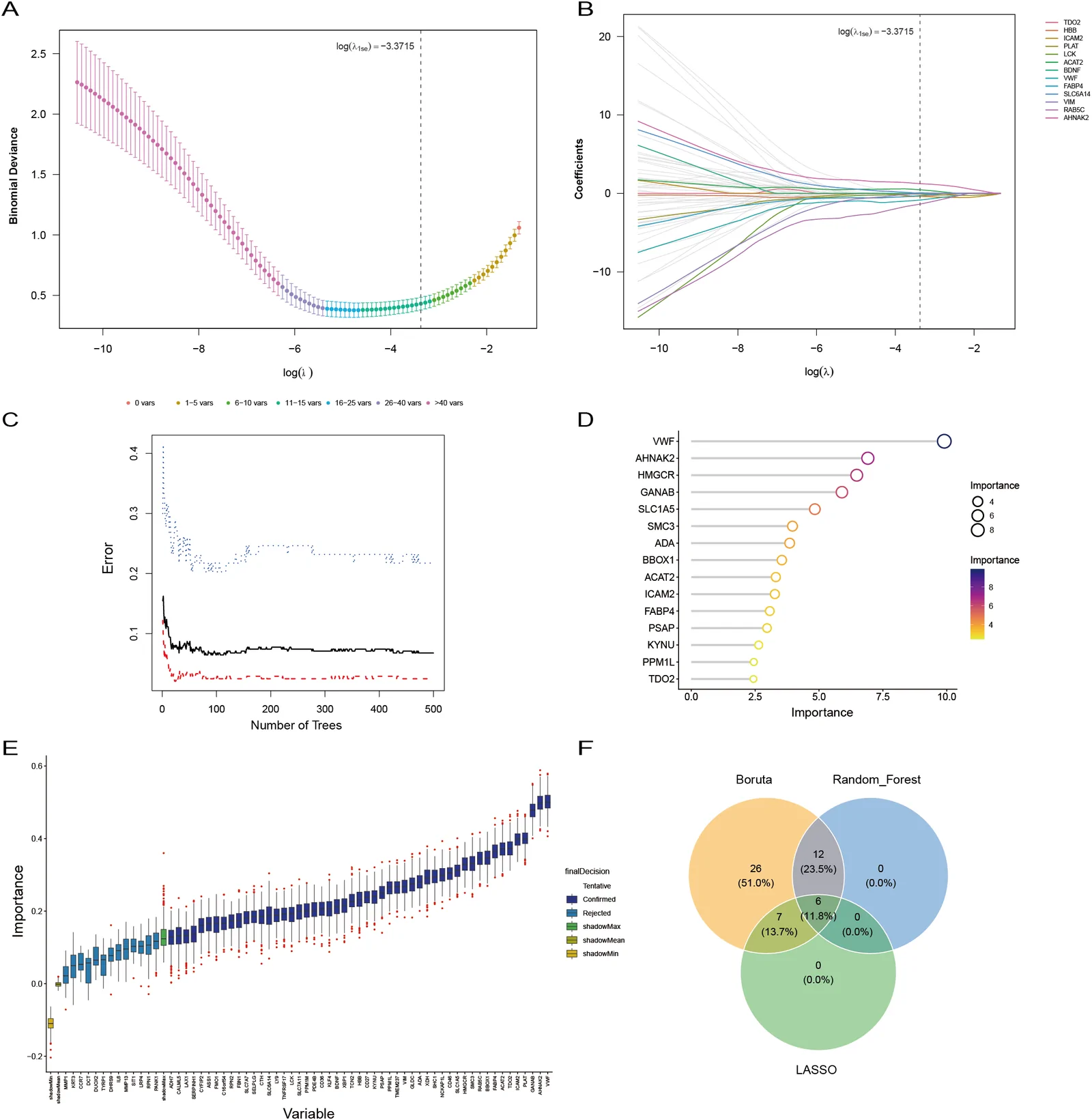
Screening of potential diagnostic biomarkers. **(A)** Cross-validation curve plot. The x-axis shows log(λ), the LASSO regularization parameter controlling feature selection. The y-axis shows binomial deviance, with lower values indicating better model fit. Red dots represent mean deviance at each log(λ), and gray error bars indicate standard error. **(B)** Gene coefficient plot. The plot shows the trajectories of candidate gene coefficients across log(λ) values during LASSO-based feature selection. **(C)** Random forest error curve plot. The x-axis shows the number of trees, and the y-axis shows the model error rate. The curves represent the overall and class-specific classification errors. As the number of trees increases, the error decreases and stabilizes, indicating stable classification performance. **(D)** Random forest gene importance plot showing the 15 highest-ranked genes among the 18 RF-selected genes. The x-axis shows the gene importance score, and the y-axis lists candidate genes. The position, size, and color of each circle reflect gene importance, with higher scores indicating greater contribution to model classification. **(E)** Boruta-based identification of candidate genes. Colors indicate the final decision for each variable. **(F)** Venn diagram showing the overlap among the 13 LASSO-selected genes, 18 RF-selected genes, and 51 Boruta-confirmed genes. Six genes were shared by all three methods.

The expression patterns of these six core candidate genes were then evaluated in the GSE16134 training dataset (**Figure 4A**), the independent validation dataset GSE223924 (**Figure 4B**), and the GSE10334 reproducibility cohort (**Supplementary Figure S1A**). All six genes showed significant PD-CT expression differences with concordant directions of change across all three datasets (Wilcoxon rank-sum test, all P < 0.05). TDO2, ICAM2, VWF, and FABP4 were consistently higher in PD samples, whereas ACAT2 and AHNAK2 were consistently lower in PD samples.

**Figure 4:**
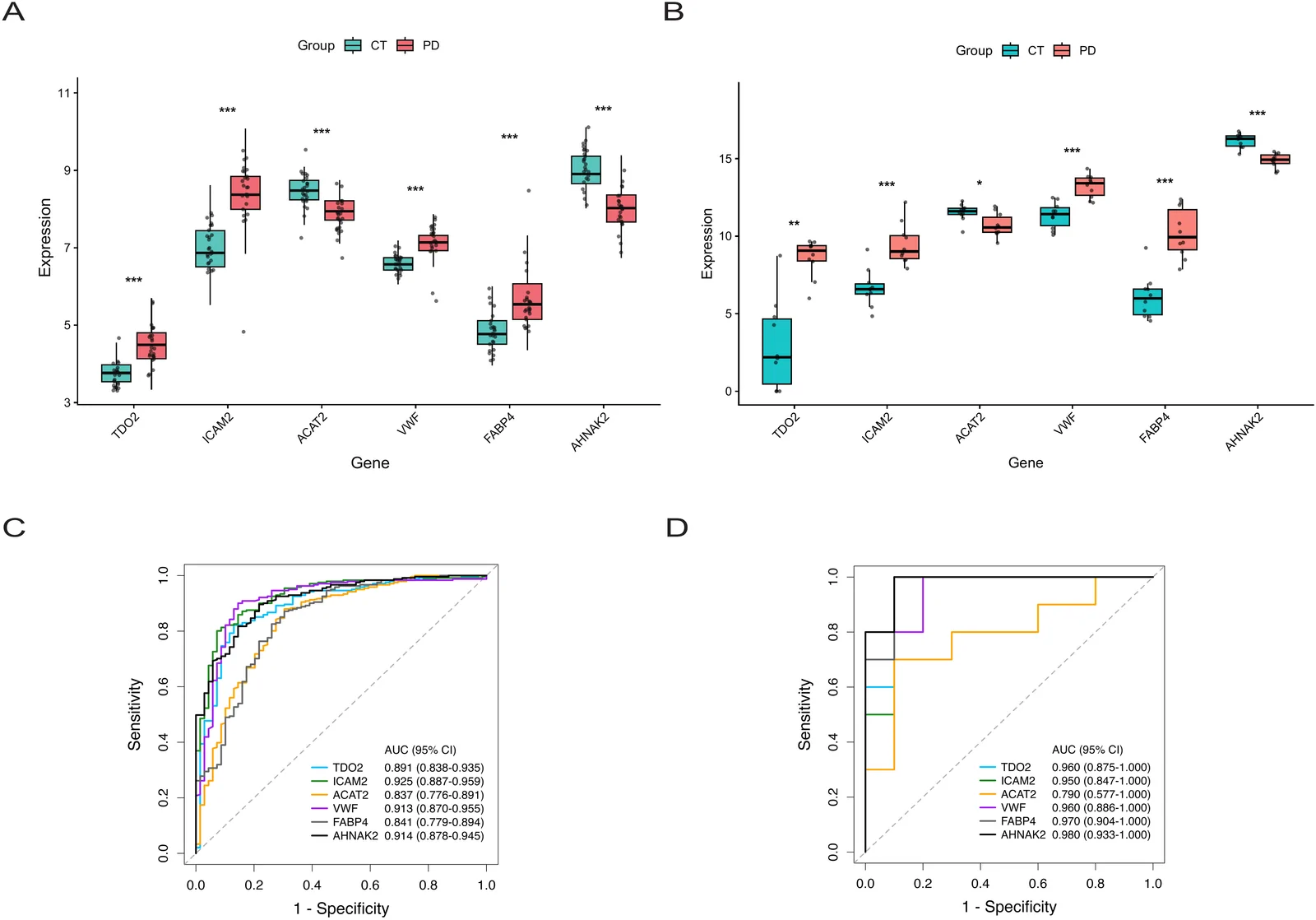
Identification of TDO2, ICAM2, ACAT2, VWF, FABP4 and AHNAK2 as biomarkers. **(A)** Expression difference analysis of the biomarkers between the PD and CT groups in the training set GSE16134. **(B)** Expression difference analysis of the biomarkers between the PD and CT groups in the validation set GSE223924. *P < 0.05, **P < 0.01, ***P < 0.001. **(C)** ROC curves of the biomarkers in the training dataset GSE16134. **(D)** ROC curves of the biomarkers in the validation dataset GSE223924.

Consistent with these expression differences, individual-gene ROC analyses yielded AUCs of at least 0.79 for all six core candidate genes in GSE16134 (**Figure 4C**), GSE223924 (**Figure 4D**) and GSE10334 (**Supplementary Figure S1B**). On the basis of their reproducible expression patterns, these six genes were retained as diagnostic biomarkers for subsequent model construction and biological characterization.

### 3.4 The six-gene nomogram showed stable diagnostic performance in the training dataset

A multivariable logistic regression nomogram was constructed using TDO2, ICAM2, ACAT2, VWF, FABP4 and AHNAK2. In the same gene order, the regression coefficients were 0.5754, 0.5120, −1.1661, 3.1253, 0.9897 and −1.9095; the corresponding ORs were 1.778, 1.669, 0.312, 22.766, 2.691 and 0.148, and VIFs were 1.700, 1.978, 1.158, 1.977, 1.148 and 1.812. The intercept was −5.9058. ORs represent a one-unit increase in the expression values used for model fitting, holding the other predictors constant. In the GSE16134 training dataset, the model achieved an apparent AUC of 0.956 (95% CI: 0.918–0.985; **Figure 5A**). The calibration curve was close to the ideal reference line, and DCA indicated positive net benefit across a range of threshold probabilities (**Figures 5B, C**). The corresponding nomogram, which converts the summed scores of the six biomarkers into a predicted probability of PD, is shown in **Figure 5D**.

**Figure 5:**
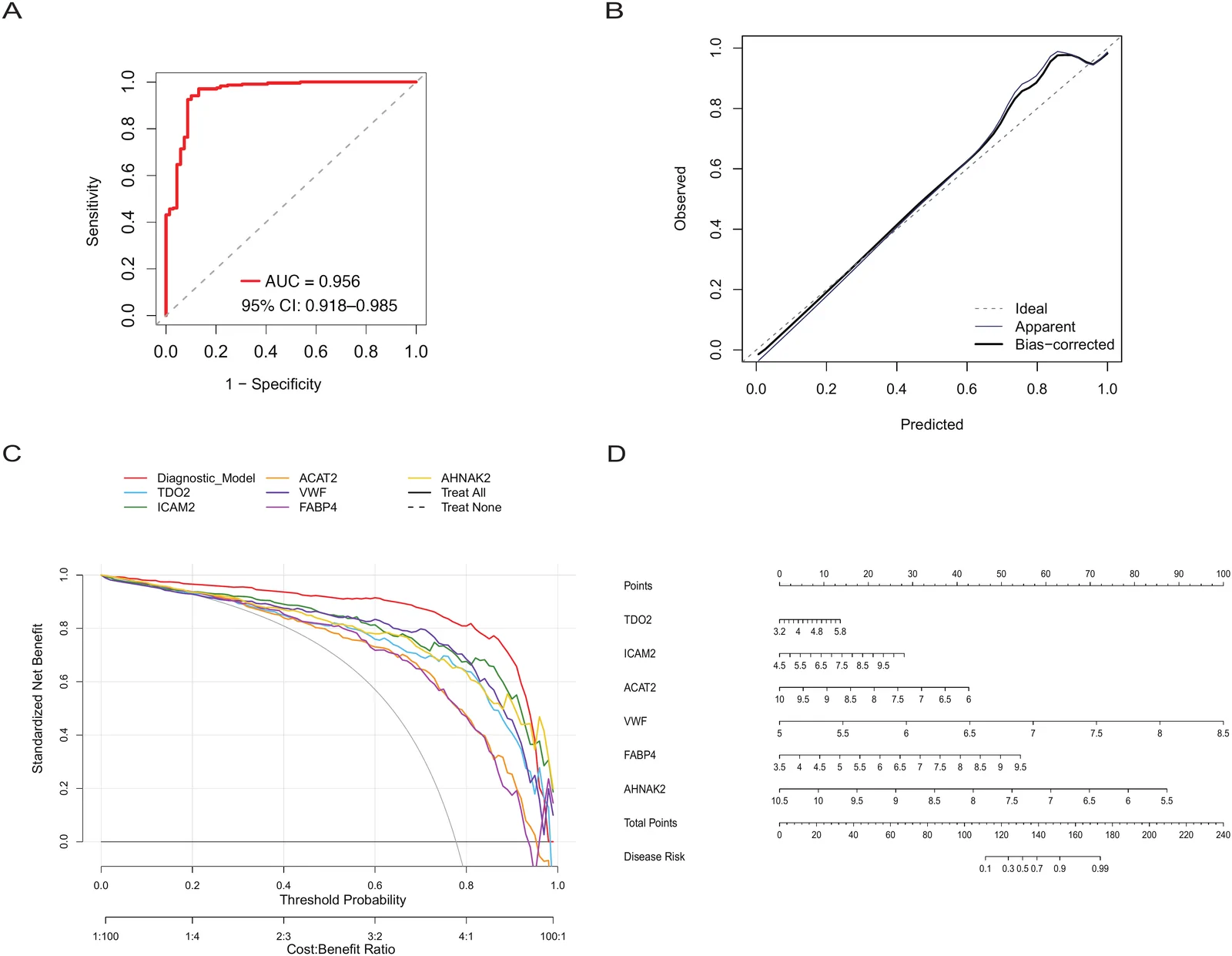
Construction and evaluation of the nomogram model. **(A)** ROC curve of the nomogram model in the training set GSE16134 (AUC = 0.956). **(B)** Calibration curve of the nomogram model in the training set GSE16134. **(C)** DCA analysis of the nomogram model in the training set GSE16134. **(D)** Nomogram developed based on core genes in the training set GSE16134.

The model achieved AUCs of 0.937 (95% CI: 0.894–0.976) in the GSE10334 reproducibility cohort and 0.990 (95% CI: 0.962–1.000) in the independent validation cohort GSE223924. Using the same training-derived cutoff, sensitivity and specificity were 95.6% and 85.9% in GSE10334, and 40.0% and 100.0% in GSE223924, respectively. Despite its high discrimination, the model showed calibration deviations in GSE223924. ROC curves with AUCs and 95% confidence intervals, calibration curves and decision curves for both cohorts are presented in **Supplementary Figure S2**.

### 3.5 Interaction network analysis of the six diagnostic biomarkers

Protein-level and functional association networks were constructed to examine relationships among the six diagnostic biomarkers and related genes. STRING analysis generated a PPI network containing TDO2, AHNAK2, VWF, ICAM2 and FABP4 together with their interacting proteins; ACAT2 retained no interaction above the applied confidence threshold and therefore does not appear in the network (**Supplementary Figure S3A**). VWF, ICAM2, and FABP4 were connected with multiple related proteins, including ASGR1, CLEC4M, ESM1, ICAM4, and ESAM. These network partners were mainly associated with cell adhesion, vascular endothelial function, and immune regulation. GeneMANIA analysis further showed that the six diagnostic biomarkers were linked to functionally related genes through physical interactions, shared protein domains, and gene-gene functional associations (**Supplementary Figure S3B**).

### 3.6 GSEA of six diagnostic biomarkers

GSEA was performed separately for TDO2, ICAM2, ACAT2, VWF, FABP4, and AHNAK2 to characterize pathways associated with each diagnostic biomarker. Cytokine-cytokine receptor interaction was detected in the enrichment results for all six biomarkers. TDO2 enrichment covered chemokine signaling, cytokine-cytokine receptor interaction and hematopoietic cell lineage (**Figure 6A**). For ICAM2 the hits were B-cell receptor signaling, lysosome and N-glycan biosynthesis (**Figure 6B**). ACAT2 returned B-cell receptor signaling, chemokine signaling and cytokine-cytokine receptor interaction (**Figure 6C**). VWF mapped to cell adhesion molecules and leukocyte transendothelial migration (**Figure 6D**). For FABP4 the enriched set comprised B-cell receptor signaling, N-glycan biosynthesis and protein export (**Figure 6E**). AHNAK2 gave the same three pathways as TDO2 (**Figure 6F**). Together, these enrichment profiles converged on cytokine and chemokine signaling, immune-cell activation, cell adhesion, and protein-processing programs.

**Figure 6:**
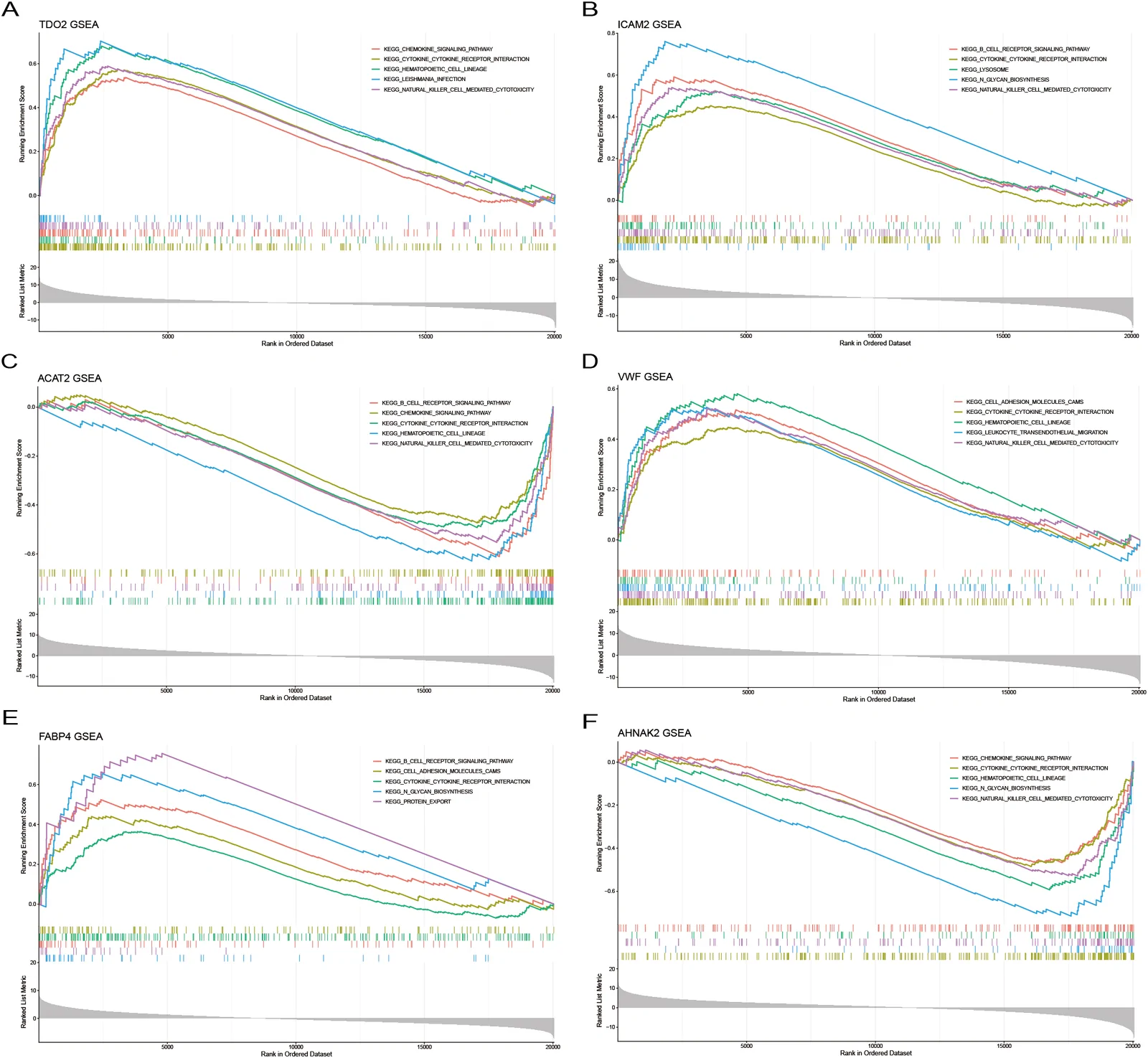
GSEA of diagnostic biomarkers. **(A)** GSEA of TDO2 biomarker. **(B)** GSEA of ICAM2 biomarker. **(C)** GSEA of ACAT2 biomarker. **(D)** GSEA of VWF biomarker. **(E)** GSEA of FABP4 biomarker. **(F)** GSEA of AHNAK2 biomarker.

### 3.7 Diagnostic biomarkers correlated with immune infiltration and inflammatory mediators

Immune infiltration analysis showed two main biomarker-associated patterns. In CIBERSORT analysis, ACAT2 and AHNAK2 showed similar correlation profiles, with positive correlations with resting dendritic cells, resting mast cells, follicular helper T cells, and M1 macrophages, and negative correlations with plasma cells. By contrast, FABP4, ICAM2, TDO2, and VWF were positively correlated with plasma cells and activated memory CD4+ T cells and negatively correlated with resting dendritic cells. ICAM2 showed the strongest positive correlation with plasma cells and was negatively correlated with resting dendritic cells, follicular helper T cells, and M1 macrophages (**Figure 7A**). MCP-counter analysis showed a similar separation: ACAT2 and AHNAK2 were negatively correlated with B lineage, monocytic lineage, endothelial cells, cytotoxic lymphocytes, and T cells, whereas FABP4, ICAM2, TDO2, and VWF were mostly positively correlated with these immune or stromal populations (**Figure 7C**).

**Figure 7:**
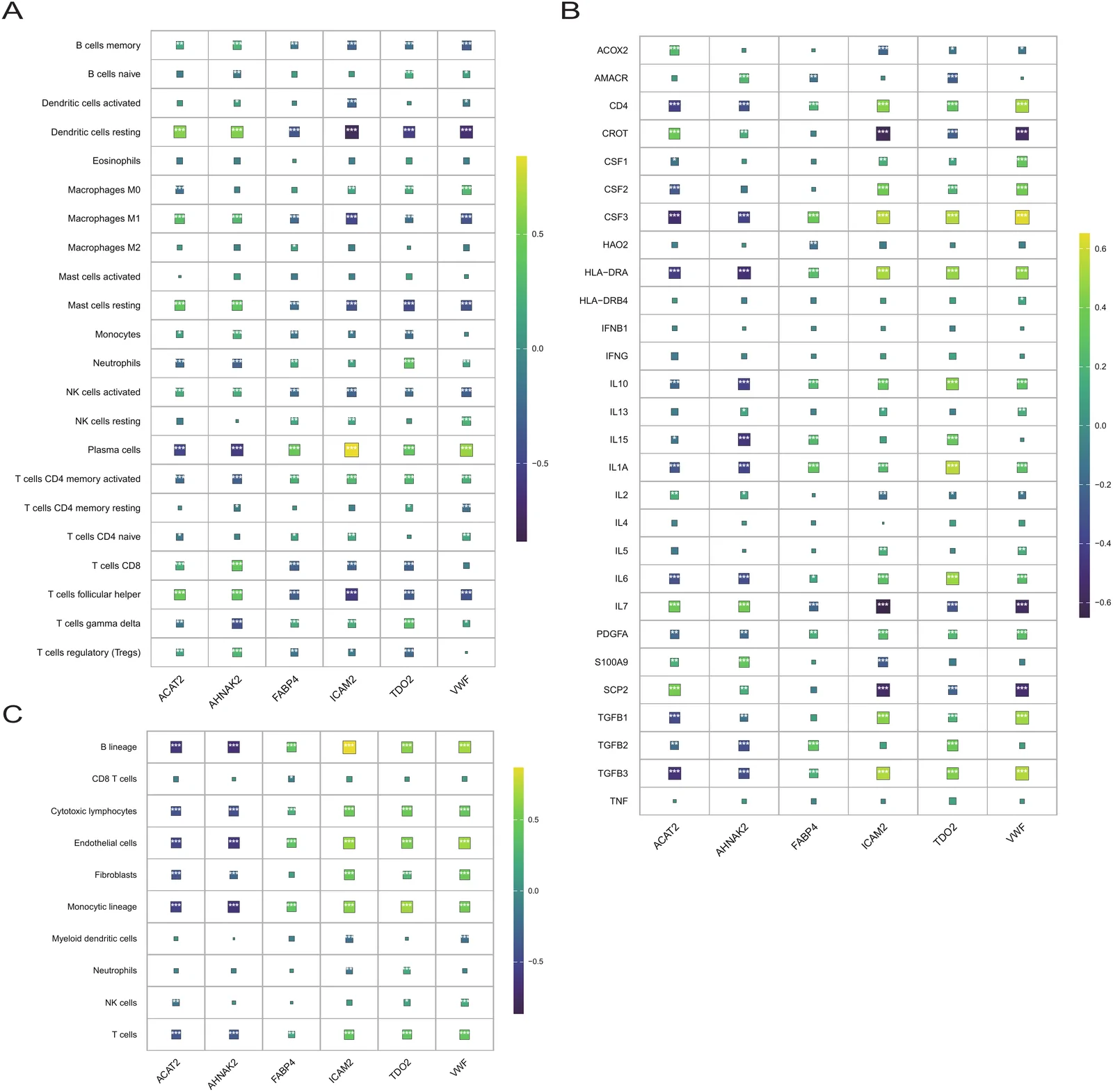
Correlations between biomarkers, immune cells and inflammatory factors. **(A)** Heatmap illustrating immune cell infiltration based on the CIBERSORT algorithm. **(B)** Spearman correlation analysis between inflammation-related genes and the six diagnostic biomarkers. **(C)** Heatmap depicting immune cells infiltration based on the MCP-counter algorithm. In panels **(A–C)**, P denotes the Benjamini–Hochberg-adjusted P value, with adjustment performed separately across all correlation tests within each panel. *P < 0.05, **P < 0.01, and ***P < 0.001.

The six diagnostic biomarkers also correlated with multiple inflammatory mediators. ACAT2 and AHNAK2 were positively correlated with IL7 and S100A9 and negatively correlated with CSF3, HLA-DRA, TGFB1, TGFB3, IL10, and IL15. FABP4 was positively correlated with CSF3, IL1A, TGFB2, HLA-DRA, and IL15. ICAM2, TDO2, and VWF showed positive correlations with several inflammation-related genes, especially CSF3, IL1A, IL6, IL10, HLA-DRA, TGFB1, and TGFB3 (**Figure 7B**). These analyses identified associations between biomarker expression, inferred immune or stromal cell profiles, and inflammatory gene expression. Biomarker expression correlated with estimated immune-cell abundance, with these associations potentially reflecting differences in tissue cell composition across the samples.

### 3.8 Identification of three molecular subtypes in PD

Consensus clustering was performed in 241 PD samples using the expression profiles of FABP4, ICAM2, VWF, ACAT2, AHNAK2, and TDO2. The consensus matrix, the delta area plot and the cumulative distribution function curve together resolved three subtypes among the PD samples: C1 (n = 115), C2 (n = 82) and C3 (n = 44) (**Figures 8A–C**). PCA showed distinct distribution patterns among the three subtypes (**Figure 8D**). All six diagnostic biomarkers differed significantly among the subtypes (all P < 0.001). FABP4 peaked in C3; ICAM2, VWF and TDO2 in C2; ACAT2 and AHNAK2 in C1 (**Figure 8E**). These results characterized three expression-based subgroups of PD samples with distinct amino acid metabolism-related biomarker profiles.

**Figure 8:**
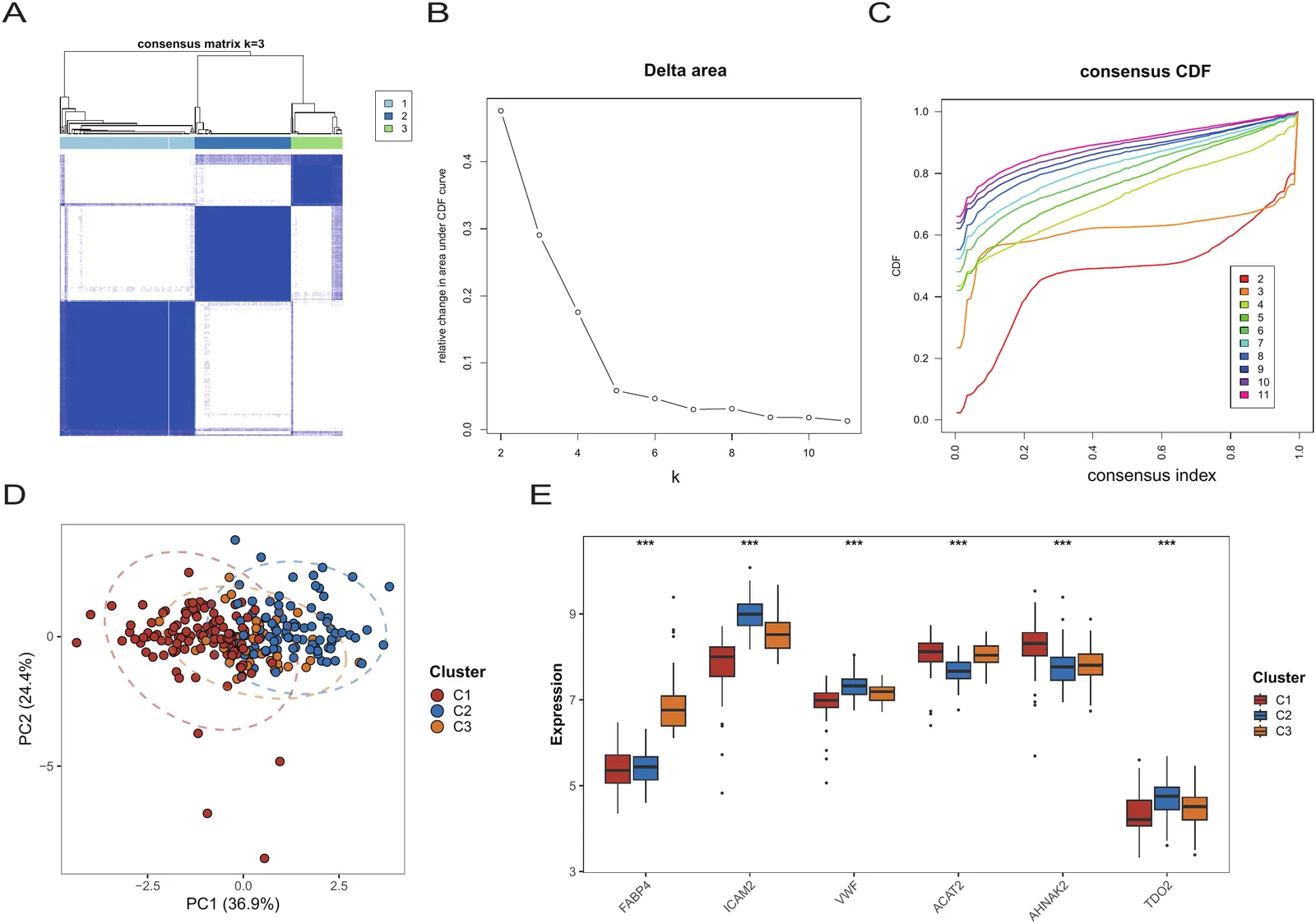
Consensus clustering analysis for PD. **(A)** Heatmap: consensus clustering results matrix for PD samples. **(B)** Delta plot of consensus clustering analysis. **(C)** Consensus cumulative distribution function analysis. **(D)** PCA plot: three subtypes of PD. **(E)** Comparison chart of biomarkers in PD between three subtypes. ***P < 0.001.

### 3.9 PD molecular subtypes showed distinct immune and inflammatory profiles

The three PD molecular subtypes differed in immune infiltration and inflammatory gene expression. MCP-counter analysis showed that C2 and C3 had higher abundance of T cells, cytotoxic lymphocytes, B lineage, monocytic lineage, and endothelial cells than C1, with most of these cell populations showing the highest abundance in C2. C3 showed relatively low neutrophil abundance, whereas C2 showed higher fibroblast abundance (**Supplementary Figure S4A**). CIBERSORT analysis further showed higher plasma cell infiltration in C2 and C3. C1 showed higher infiltration of CD8 T cells, follicular helper T cells, M1 macrophages, resting and activated dendritic cells, and activated mast cells. C3 showed higher proportions of activated memory CD4+ T cells and gamma-delta T cells but a lower proportion of neutrophils (**Supplementary Figure S4B**).

Inflammation-related gene expression also differed across the three subtypes. IL6, TGFB1, IL10, TGFB3, IL13, CD4, CSF3, and CSF2 were more highly expressed in C2. TGFB2, IL2, IL7, SCP2, CROT, and ACOX2 were more highly expressed in C1. HLA-DRA was more highly expressed in C2 and C3, whereas S100A9 showed the highest expression in C3 (**Supplementary Figure S4C**). These findings showed subtype-specific immune and inflammatory features among PD samples.

### 3.10 WGCNA and enrichment analysis of PD molecular subtypes

WGCNA was then applied to characterize co-expression modules associated with the expression-based subgroups. After dynamic tree cutting and merging of similar modules, 12 modules remained (**Figures 9A, C**). Among the module-trait correlations, MEdarkgreen was the most strongly anti-correlated with C1 (r = −0.70, P = 1 × 10^−^³^6^) and the most strongly correlated with C2 (r = 0.67, P = 3 × 10^−^³²) (**Figure 9B**). MEdarkgreen was therefore selected as the subtype-associated module for downstream enrichment analysis.

**Figure 9:**
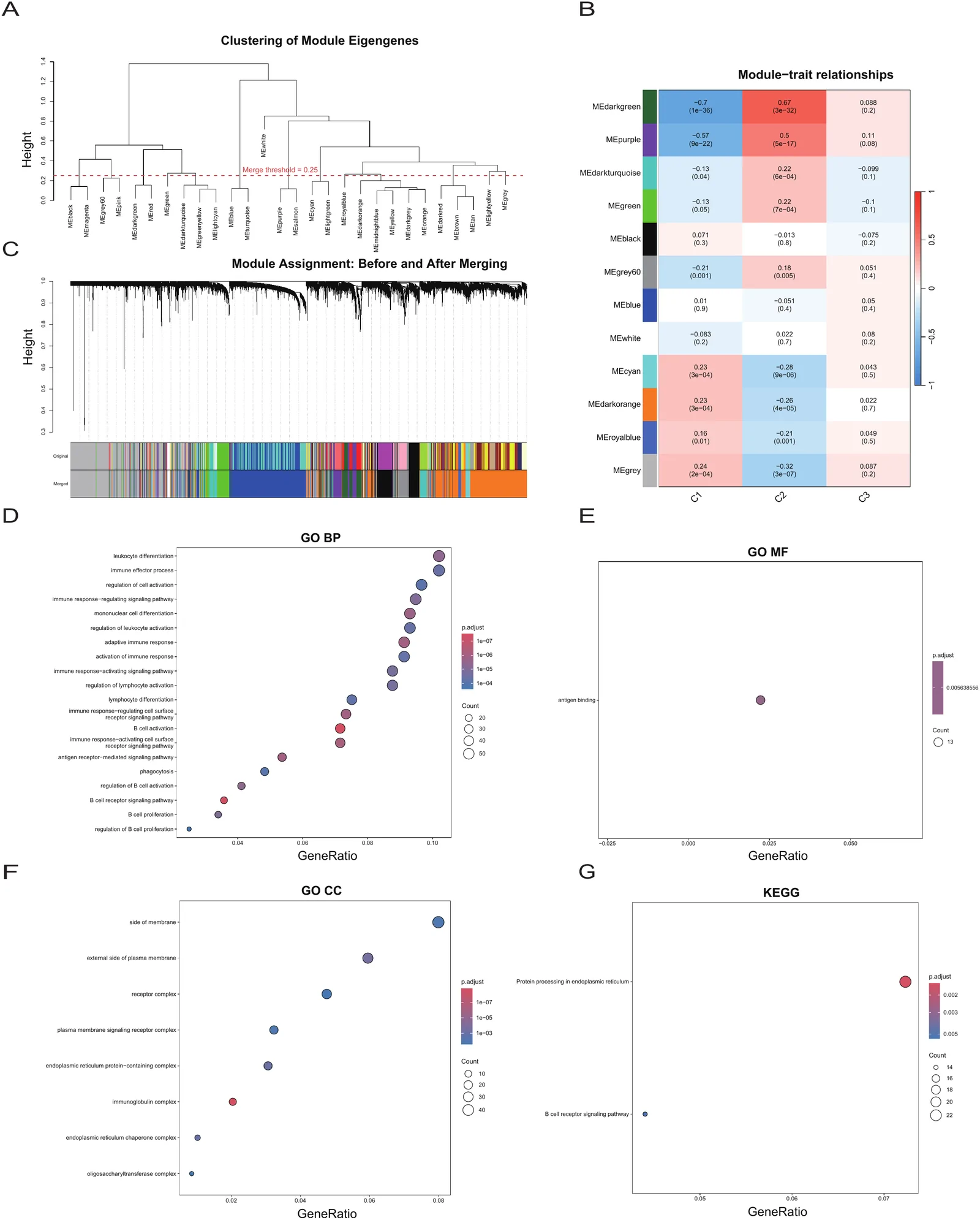
Identification of key modules through WGCNA. **(A)** Clustering dendrogram of genes based on topological overlapping. **(B)** Heatmap of the correlation between module eigengenes and three clusters. **(C)** Gene dendrogram with module assignments before and after module merging. **(D-G)** Functional enrichment analysis of genes in the MEdarkgreen module, including GO biological process (BP), GO molecular function (MF), GO cellular component (CC), and KEGG pathway analyses.

GO-BP terms for MEdarkgreen genes covered leukocyte differentiation, immune effector processes, regulation of immune cell activation, adaptive immune response, activation and proliferation of B cells, the B-cell receptor signaling pathway, and phagocytosis (**Figure 9D**). GO-MF terms were mainly enriched in antigen binding (**Figure 9E**). GO-CC terms involved the side of membrane, external side of plasma membrane, the receptor complex, the immunoglobulin complex, and endoplasmic reticulum protein-containing and chaperone complexes (**Figure 9F**). KEGG analysis showed enrichment in two pathways: protein processing in the endoplasmic reticulum, and the B-cell receptor signaling pathway (**Figure 9G**).

### 3.11 scRNA-seq annotation identified 11 cell types

GSE164241 scRNA-seq data were analyzed to localize PD-associated biomarker activity at cellular resolution. After quality control, cells with 200–4,000 detected genes and a mitochondrial gene percentage below 20% were retained (**Figure 10A**). Following ambient RNA correction, 3,589 doublets (4.07%) were excluded (**Figures 10B, C**). Normalization identified 2,000 highly variable genes, with the top 10 variable genes shown in **Figure 10D**. The first 20 principal components were used for batch correction, dimensionality reduction, and clustering (**Figures 10E, F**). The final annotated dataset comprised 84,574 cells, including 50,666 cells from healthy controls (CT) and 33,908 cells from periodontitis samples (PD). Cluster annotation based on marker genes identified 11 cell populations: B cells, endothelial cells, epithelial cells, fibroblasts, lymphatic endothelial cells, mast cells, melanocytes, mural cells, myeloid cells, plasma cells, and T cells (**Figures 10G, H**). Marker gene dot plots supported the annotation of these cell populations (**Figure 10I**).

**Figure 10:**
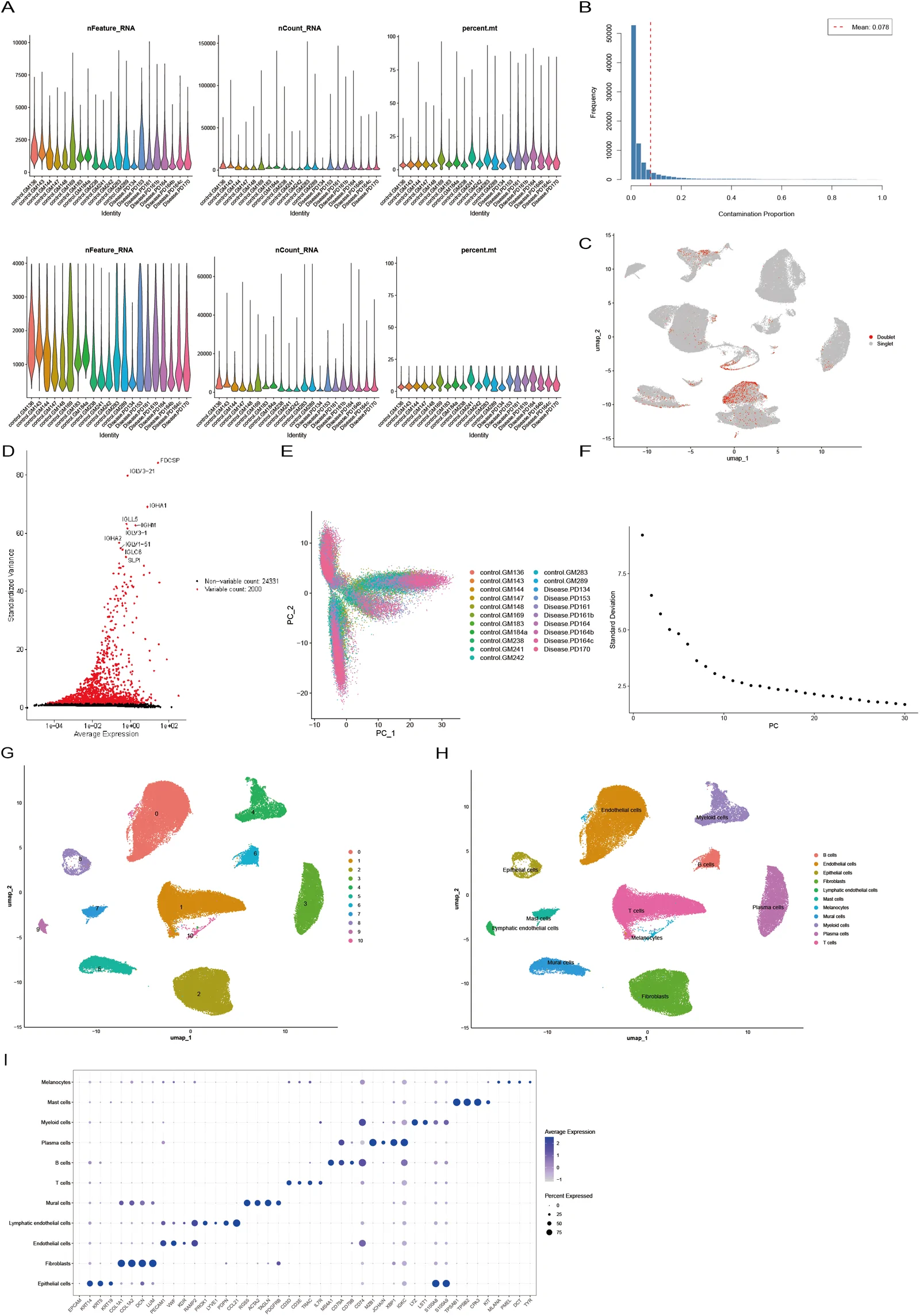
Cell type annotation analysis of scRNA-seq. **(A)** Quality control metrics before and after filtering (number of genes, total number of mRNA molecules, proportion of mitochondrial genes). **(B)** Distribution of estimated contamination proportions across cells. **(C)** UMAP visualization showing singlet and doublet classification. **(D)** Screening of highly variable genes. **(E)** PCA plot showing cell distribution across the first two principal components. **(F)** Elbow plot showing the standard deviation of principal components. **(G)** UMAP cell clustering plot. **(H)** UMAP plot of annotated cell types. **(I)** Bubble plot showing marker gene expression for cell type annotation.

### 3.12 Identification of endothelial cells as the key cell type in PD

Cell composition and Ro/e analyses showed group-level differences among the 11 annotated cell populations. Compared with the CT group, T cells, plasma cells, and myeloid cells were relatively enriched in the PD group, whereas endothelial cells, fibroblasts, and lymphatic endothelial cells showed lower relative proportions among the recovered cells (**Figure 11A**; **Supplementary Figure S5A**). Within-cell-type differential expression analysis showed that endothelial cells and fibroblasts were the only cell types in which all six diagnostic biomarkers differed significantly between PD and CT cells (Wilcoxon rank-sum test, P < 0.05; **Figure 11D**).

**Figure 11:**
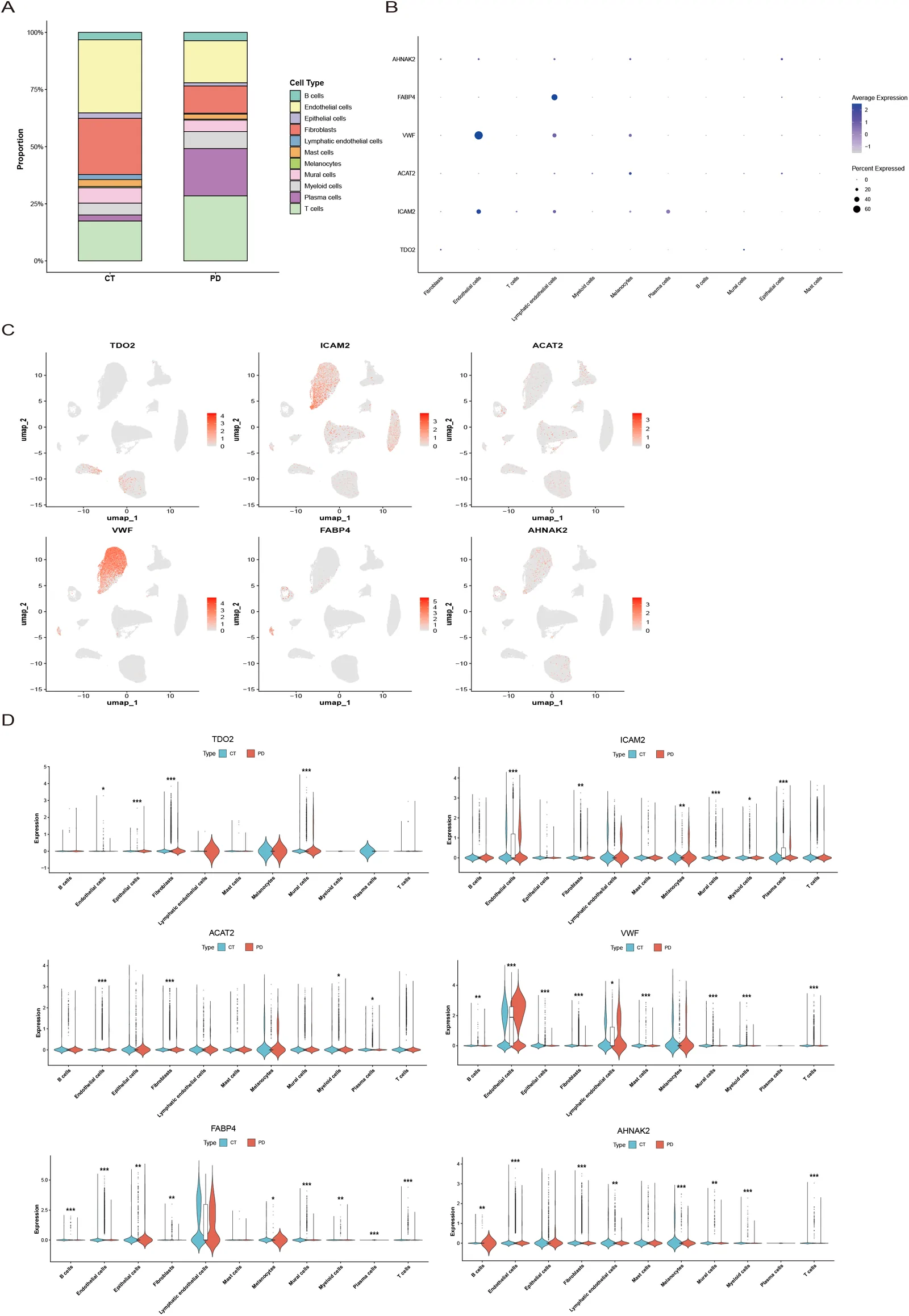
Identification of key cells and analysis of related characteristics. **(A)** Stacked bar plot showing the proportions of annotated cell types in the two groups **(B)** Bubble plot showing the average expression level and percentage of expressing cells for each diagnostic biomarker across the 11 annotated cell types. **(C)** UMAP distribution of biomarkers in key cells. Color indicates gene expression levels, with darker shades representing higher expression. Gray indicates low or absent expression of the gene in the corresponding cell. **(D)** Violin plots comparing the expression levels of candidate biomarkers between the control and PD groups across annotated cell types. *P < 0.05, **P < 0.01, ***P < 0.001.

The distribution of individual diagnostic biomarkers further highlighted endothelial cells. VWF and ICAM2 were mainly expressed in endothelial cells and showed higher expression in endothelial cells than in most other annotated cell types (**Figures 11B, C**). Endothelial cells also carried the highest diagnostic biomarker ModuleScore among the candidate key cell types; among the remaining populations, only lymphatic endothelial cells reached comparable scores (**Supplementary Figure S5B**). Although fibroblasts also showed significant between-group differences for all six biomarkers, their diagnostic biomarker ModuleScore was substantially lower than that of endothelial cells, and none of the six biomarkers showed preferential expression in fibroblasts. Integrating cell composition, single-gene differential expression, cell-type specificity of individual biomarkers, and the diagnostic biomarker ModuleScore, endothelial cells were therefore identified as the key cell type.

### 3.13 Specific communications among cell types

CellChat analysis showed different intercellular communication patterns between the PD and CT groups. Compared with the CT group, the predicted number of cell-cell communications and overall communication strength increased in the PD group. Endothelial cells also showed increased incoming, outgoing, and total communication scores in the CellChat-inferred network (**Figures 12A–D**).

**Figure 12:**
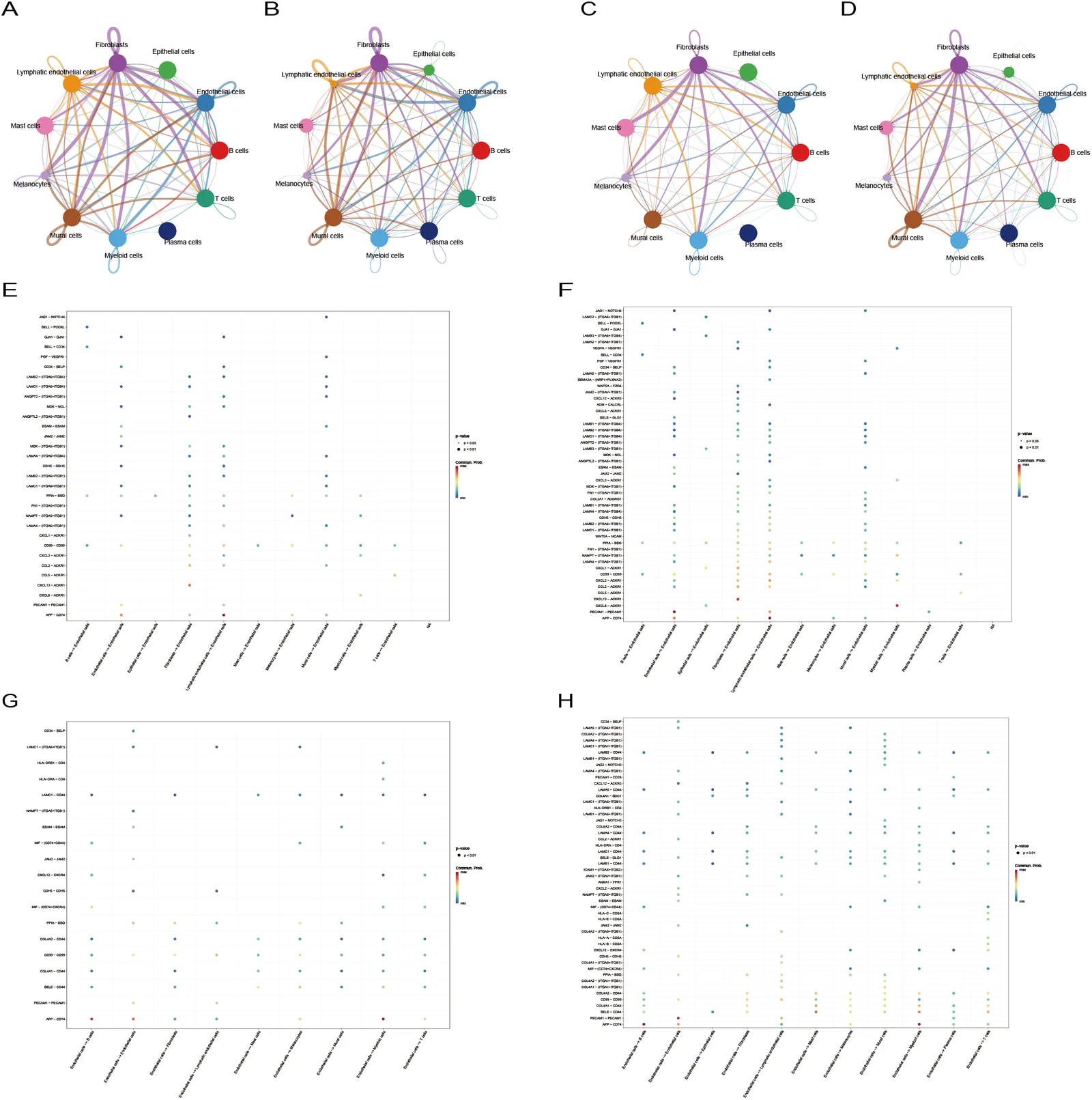
Analysis of specific communications among cell types. **(A)** Cell communication number network of 11 cell types in the CT group. **(B)** Cell communication number network of 11 cell types in the PD group. **(C)** Cell communication weight network of 11 cell types in the control group. **(D)** Cell communication weight network of 11 cell types in the PD group. Each cell type is represented by a node, where the node size reflects the number of communications the cell type is involved in, and the color shade represents the communication intensity. The connections between cell types denote communication relationships, and the thickness of the lines corresponds to the number of communications. **(E)** Bubble plot of ligand-receptor pairs in which endothelial cells act as signal receivers in the CT group. **(F)** Bubble plot of ligand-receptor pairs in which endothelial cells act as signal receivers in the PD group. **(G)** Bubble plot of ligand-receptor pairs in which endothelial cells act as signal senders in the CT group. **(H)** Bubble plot of ligand-receptor pairs in which endothelial cells act as signal senders in the PD group. Each dot in the figure represents a ligand-receptor pair in specific intercellular communication. The color shade of the dot indicates the communication probability, and the size of the dot reflects the statistical significance (P-value).

Ligand-receptor analysis showed enhanced endothelial-to-T-cell communication in the PD group. These interactions included SELE-CD44, COL4A1-CD44, COL4A2-CD44, CXCL12-CXCR4, and HLA-B/HLA-A/HLA-E/HLA-C-CD8A. Endothelial cells also showed strong APP-CD74 communication with B cells and myeloid cells. In addition, CellChat predicted endothelial-to-endothelial PECAM1-PECAM1 interactions and incoming interactions from myeloid cells, fibroblasts, and lymphatic endothelial cells involving CXCL8-ACKR1, CXCL13-ACKR1, and APP-CD74 (**Figures 12E–H**). These results showed that endothelial cells occupied an active communication position in the PD intercellular signaling network.

### 3.14 Pseudotime trajectory analysis of endothelial cells

Endothelial cells were extracted and subjected to reclustering and pseudotime analysis. At a resolution of 0.2, endothelial cells were divided into six subgroups, labeled 0-5 (**Figure 13A**). Trajectory analysis showed a continuous endothelial state trajectory with two branch points along pseudotime. Subgroups 2, 3, 4, and 5 were mainly distributed in early trajectory regions and across different branches, whereas subgroups 0 and 1 were more frequent at later pseudotime stages (**Figures 13B–D**).

**Figure 13:**
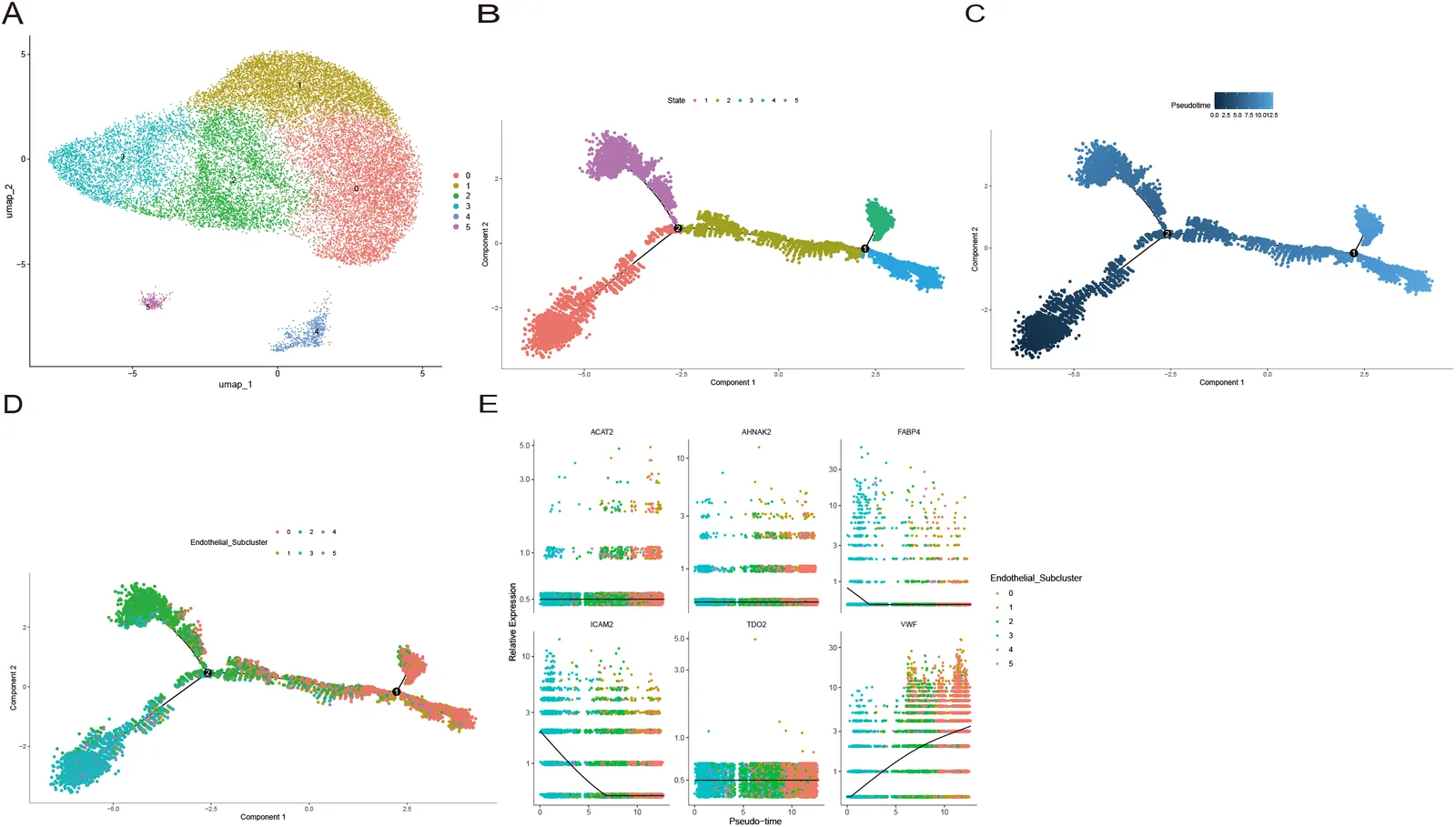
Pseudotime analysis of Endothelial cells. **(A)** Clustering plot of Endothelial cells subclusters. **(B)** Monocle 2 pseudotime trajectory of endothelial cells colored by cell state. **(C)** The same trajectory colored by pseudotime. **(D)** The same trajectory colored by endothelial subcluster. **(E)** Dynamic expression of the six diagnostic biomarkers (ACAT2, AHNAK2, FABP4, ICAM2, TDO2 and VWF) along pseudotime; points are colored by endothelial subcluster and black lines show the fitted expression trend.

The six diagnostic biomarkers showed different dynamic expression patterns along pseudotime. ICAM2 and FABP4 were mainly expressed at early pseudotime stages and gradually decreased as endothelial states progressed. VWF gradually increased along pseudotime and was mainly expressed in late endothelial cells. AHNAK2 also showed pseudotime-related variation, whereas ACAT2 and TDO2 showed relatively stable overall trends (**Figure 13E**). These results showed that diagnostic biomarker expression varied along the inferred endothelial trajectory.

### 3.15 Experimental validation of diagnostic biomarker expression in clinical samples

Five of the six diagnostic biomarkers were selected for experimental validation; AHNAK2 was not validated because it showed the smallest effect size across the three bulk cohorts and, unlike the other five biomarkers, has no reported functional link to periodontitis; experimental validation was therefore prioritized for the five biomarkers with clearer mechanistic support. A total of 40 gingival tissue specimens from 20 patients with periodontitis and 20 healthy controls were included in the clinical tissue analyses. H&E staining, RT-qPCR, and immunohistochemistry each included specimens from 10 donors per group.

H&E staining showed more pronounced inflammatory cell infiltration and tissue destruction in periodontitis samples than in healthy control samples (**Figures 14A, B**). These findings were consistent with the characteristic histopathological features of periodontitis.

**Figure 14:**
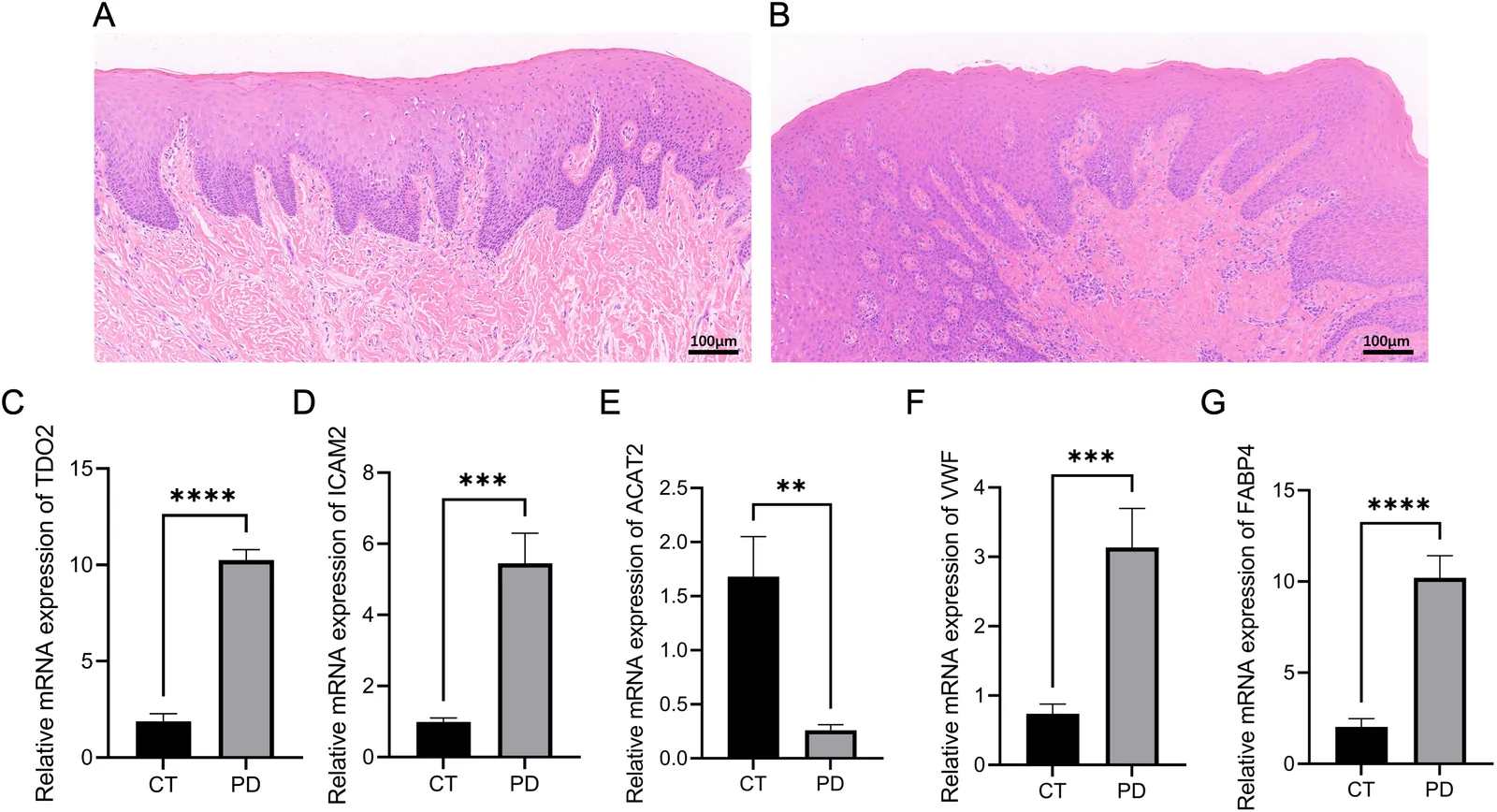
Validation of bioinformatics analysis based on clinical samples. **(A, B)** Representative hematoxylin-eosin staining of gingival tissue from the CT **(A)** and PD **(B)** groups (n=10). **(C-G)** RT-qPCR analysis of TDO2 **(C)**, ICAM2 **(D)**, ACAT2 **(E)**, VWF **(F)** and FABP4 **(G)** relative mRNA expression in gingival tissues. **P < 0.01, ***P < 0.001, ****P < 0.0001 (n=10).

RT-qPCR showed significantly higher mRNA levels of TDO2, ICAM2, VWF, and FABP4 and a lower ACAT2 level in periodontitis samples than in healthy control samples (all P < 0.01; **Figures 14C–G**). Consistently, immunohistochemical analysis showed stronger staining for TDO2, ICAM2, VWF, and FABP4 and weaker staining for ACAT2 in periodontitis samples than in healthy control samples (**Figure 15A**). Quantification of the mean optical density confirmed significant between-group differences in the immunoreactivity of all five proteins (**Figures 15B–F**). These directions of change were consistent with those observed in the GSE16134 training dataset and the GSE10334 validation dataset. Together, these findings provided clinical-sample support for five of the six candidate diagnostic biomarkers identified by the bioinformatics analysis.

**Figure 15:**
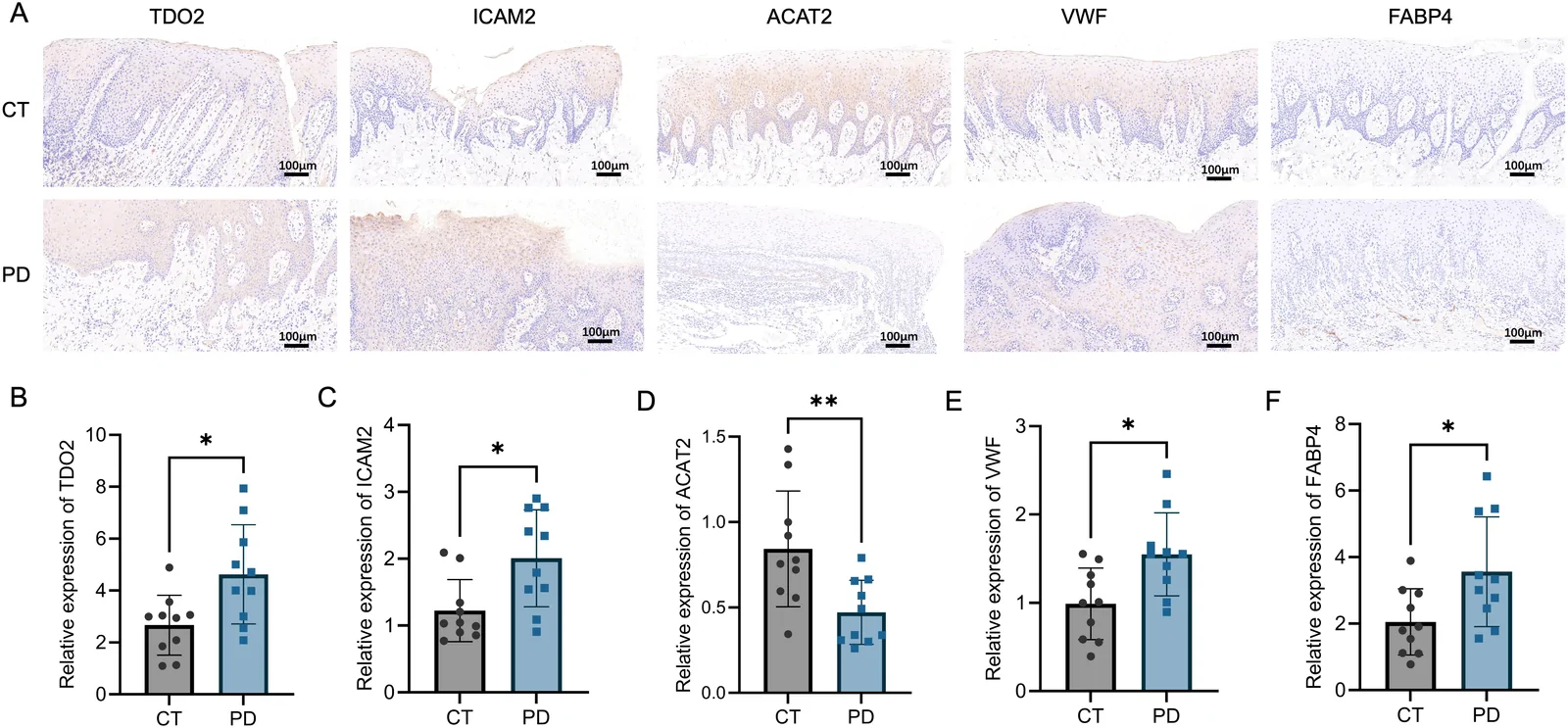
Validation of diagnostic biomarker expression at the protein level in clinical samples. **(A)** Representative immunohistochemical staining of TDO2, ICAM2, ACAT2, VWF, and FABP4 in gingival tissues from the CT and PD groups (n=10). Scale bar, 100 μm. **(B-F)** Quantification of the mean optical density of TDO2 **(B)**, ICAM2 **(C)**, ACAT2 **(D)**, VWF **(E)**, and FABP4 **(F)**. *P < 0.05, **P < 0.01.

## 4 Discussion

Periodontitis is increasingly understood not only as a dysbiosis-driven inflammatory disease but as an immune-metabolic disorder in which local nutrient availability and metabolic signaling shape the host inflammatory response (41, 42). Tissue-level expression of amino acid metabolism-related genes in periodontitis, its diagnostic value, and its cellular sources have nonetheless remained poorly defined. By integrating three bulk gingival cohorts with a single-cell dataset, machine-learning feature selection and clinical gingival sample analyses, we identified a six-gene amino acid metabolism-related signature (TDO2, ICAM2, ACAT2, VWF, FABP4 and AHNAK2) with concordant expression changes across the three bulk datasets. Clinical samples supported expression differences in five of the six genes, and single-cell analysis identified vascular endothelial cells as a key cell population associated with the signature. Together, these findings link the amino acid metabolism-related signature to endothelial alterations and immune-niche remodeling in periodontitis.

Functional annotation of the 64 amino acid metabolism-related differentially expressed genes converged on substrate transport and metabolism, immune-cell activation, and protein processing, a profile indicating a metabolism-coupled inflammatory state rather than an isolated pathway, in line with evidence that amino acid and related metabolic pathways act as hallmarks of innate immune cell activation and as drivers of macrophage polarization (43, 44). Enrichment of amino acid, organic acid and carboxylic acid transmembrane transporter activity indicates that substrate uptake and utilization are reprogrammed in inflamed gingiva, consistent with clinical metabolomic reports of elevated kynurenine metabolites and an altered KYN/TRP ratio that track with probing depth, bleeding on probing, and hypoxia (45, 46). The concurrent enrichment of leukocyte adhesion and lymphocyte activation terms, together with IL6 as the highest-degree hub of the network, places these genes within the cytokine- and adhesion-driven amplification loop that sustains periodontal inflammation (47).

Among the six biomarkers, TDO2 is of particular interest because it catalyzes the first step of tryptophan catabolism via the kynurenine pathway. Its increased expression in our study is consistent with previous reports of altered tryptophan–kynurenine metabolism in periodontal inflammation (45, 46). Previous studies have also implicated aryl hydrocarbon receptor (AhR) signaling in periodontitis (48). Downstream, kynurenine-AhR signaling shapes macrophage polarization and the Th17/Treg balance, and IDO-dependent kynurenine-AhR-NRF2 signaling mediates the bone- and inflammation-sparing effects of stromal cells in experimental periodontitis (49), whereas P. gingivalis suppresses AhR signaling and thereby promotes inflammation (50). We therefore hypothesize that TDO2 may influence periodontal immune responses through kynurenine–AhR signaling. This interpretation remains provisional because tryptophan and kynurenine concentrations, TDO2 enzymatic activity, and AhR activation were not assessed. Future metabolic and functional studies could test this hypothesis, building on the observed TDO2 upregulation in transcriptomic analyses and clinical gingival samples.

The reported functions of ICAM2, VWF, FABP4 and ACAT2 span endothelial adhesion, inflammatory signaling and lipid-related metabolism. ICAM2 is a constitutively expressed endothelial ligand of the β2 integrin LFA-1 (51) that regulates luminal neutrophil crawling and transendothelial migration (52, 53), and VWF, stored in Weibel-Palade bodies, is a canonical marker of endothelial activation that couples hemostasis to leukocyte recruitment (54, 55); circulating VWF rises with periodontal inflammatory burden and does not normalize after tooth extraction (56), consistent with the impaired endothelial function repeatedly reported in periodontitis (57). Their concurrent upregulation in PD is consistent with their reported roles in endothelial–leukocyte interactions. In gingival tissue samples, FABP4 was upregulated, whereas ACAT2 was downregulated. FABP4 has been reported to link fatty acid transport to inflammatory signaling, promote endothelial dysfunction (58), be ectopically induced in injured vascular endothelium (59), and amplify chemotactic signaling through the KLF2–FABP4–CCL20 axis (60, 61). ACAT2 encodes cytosolic acetoacetyl-CoA thiolase, which participates in cholesterol biosynthesis. Cholesterol can subsequently be esterified for storage (62), helping maintain cholesterol homeostasis (63). Taken together, these findings are consistent with reported roles in lipid metabolism and endothelial inflammation, while functional consequences await validation. AHNAK2, the sixth biomarker, lies outside this endothelial-lipid axis. It encodes a large scaffold protein involved in membrane organization, calcium signaling and cellular stress responses, including stress-induced nonclassical FGF1 secretion (64, 65). Its downregulation, together with its consistent co-behavior with ACAT2 across both deconvolution methods and across the three subtypes, is best read as attenuation of a structural and stress-adaptive program rather than as an inflammatory driver.

The amino acid metabolism-related designation reflects the annotation-derived gene set used for biomarker selection (18). TDO2 participates in tryptophan catabolism, while the other biomarkers contribute to endothelial function, immune responses, lipid metabolism and cellular stress responses. These biomarkers may help characterize vascular and immune changes accompanying periodontitis, extending the focus from amino acid metabolism to processes involved in the local inflammatory response.

Immune deconvolution and consensus clustering indicate that the signature reports on broader remodeling of the periodontal immune niche. TDO2, ICAM2, VWF and FABP4 were positively associated with plasma cells, activated memory CD4+ T cells, and B-cell, monocytic and endothelial lineages, whereas ACAT2 and AHNAK2 showed the opposite pattern, dividing the panel into an inflammation-active and a homeostatic arm. Consensus clustering resolved three subtypes, of which C2 combined high TDO2, ICAM2 and VWF expression with B-cell, plasma-cell and endothelial enrichment and with upregulation of IL6, TGFB1/3 and CSF2/3. Because the subtypes were defined by the six biomarkers themselves, the associated MEdarkgreen module characterizes rather than independently validates them. Its enrichment in B-cell activation, B-cell receptor signaling and endoplasmic reticulum protein processing is nonetheless notable, since activated B and T lymphocytes are the principal RANKL sources driving alveolar bone loss (66–68), consistent with single-cell studies placing B-cell and plasma-cell trajectories at the center of periodontal immunopathology (69).

Endothelial homeostasis is itself an amino acid-dependent process, which makes the endothelium a predicted point of convergence for this signature. Arginine-nitric oxide metabolism is a principal determinant of endothelial function, and asymmetric dimethylarginine (ADMA), an endogenous nitric oxide synthase inhibitor, suppresses nitric oxide production from L-arginine and impairs endothelium-dependent vasodilation (70); serum ADMA is elevated in chronic periodontitis and correlates with endothelial dysfunction and systemic inflammation (71). The periodontal pathogen itself targets these amino acid-linked axes, since P. gingivalis-induced endothelial dysfunction has been linked *in vivo* to altered PPARγ/Akt/eNOS and Nrf2/ARE signaling and to suppression of the AhR/IDO pathway (72, 73). Our single-cell data provide a cellular expression context for this hypothesis but do not directly assess arginine–NO pathway activity. Endothelial cells were the only compartment in which all six biomarkers differed between groups, and they carried the highest biomarker module score and the most specific expression of VWF and ICAM2. That they were nonetheless proportionally reduced by Ro/e analysis while showing the highest per-cell biomarker activity is not contradictory. Single-cell proportions are compositional (74), so the influx of T cells, plasma cells and myeloid cells necessarily compresses the fractional representation of every resident stromal population, and endothelial cells, fibroblasts and lymphatic endothelial cells were indeed all proportionally reduced. In parallel, protracted inflammation disrupts and remodels the gingival microvascular network (75), so the endothelial cells recovered from diseased tissue may form a transcriptionally activated population with a reduced relative proportion among the recovered cells. Separating compositional dilution from true endothelial loss will require absolute quantification, such as stereological vessel-density measurement or spatial transcriptomics.

Consistent with endothelial activation despite a reduced relative proportion, CellChat predicted expanded incoming and outgoing endothelial signaling in disease, including endothelial-to-T-cell adhesion and chemokine axes (SELE-CD44, CXCL12-CXCR4), APP-CD74 signaling to B and myeloid cells, and CXCL8/CXCL13-ACKR1 signals received from myeloid cells, fibroblasts and lymphatic endothelial cells. Previous studies provide biological context for the predicted ACKR1-associated interactions: venular endothelium transcytoses chemokines to the luminal surface and thereby promotes leukocyte transendothelial migration (76), and endothelial ACKR1 is itself induced by neutrophil contact (77). Pseudotime analysis described expression variation along an inferred endothelial trajectory, with ICAM2 and FABP4 expression declining and VWF expression rising along pseudotime. Previous experimental studies have also linked periodontal pathogens to endothelial dysfunction. P. gingivalis downregulates endothelial eNOS and Nrf2/phase II defenses (78) and induces mitochondrial and microvesicle-mediated endothelial dysfunction (79, 80), and tryptophan-AhR/IDO signaling modulates vascular inflammation in P. gingivalis-driven disease (81). Taken together, the inferred endothelial state changes and predicted adhesion and chemokine signaling suggest that the amino acid metabolism-related signature reflects altered endothelial–immune interactions in periodontitis. Considered alongside previous experimental evidence, these findings support the hypothesis that the vascular endothelium provides a link between amino acid-related metabolic signaling and local immune remodeling.

This study has several limitations. The analyses are predominantly transcriptomic, and although five biomarkers were validated by RT-qPCR and immunohistochemistry, their spatial localization and functional activity remain unconfirmed. Invasive gingival sampling limits the signature’s use in routine screening or repeated monitoring. These genes should therefore be considered candidate tissue biomarkers, and assays targeting them in gingival crevicular fluid, saliva, or serum require evaluation of detectability and diagnostic performance in independent cohorts before clinical use. The amino acid metabolism-related gene set is annotation-derived rather than pathway-restricted, and only TDO2 is a canonical amino acid metabolic enzyme (18), so establishing a direct metabolic role would require targeted metabolomic or flux measurement. Because several biomarkers are endothelial or myeloid in origin, the bulk signal cannot be fully separated from disease-associated shifts in cell composition, and cell-intrinsic metabolic reprogramming will require sorted or spatially resolved measurement. GSE10334 overlaps with the training cohort, and the model showed calibration deviations and low sensitivity at the training-derived cutoff in the small GSE223924 cohort despite high discrimination. Clinical expression validation was limited to 20 patients and 20 healthy individuals. Future studies should evaluate the complete six-gene model in larger, independent prospective cohorts and conduct functional perturbation studies focused on TDO2-kynurenine and endothelial arginine-NO signaling.

## 5 Conclusion

This study identified six amino acid metabolism-related candidate diagnostic biomarkers for periodontitis (TDO2, ICAM2, ACAT2, VWF, FABP4 and AHNAK2), five of which showed expression changes supported by RT-qPCR and immunohistochemistry in clinical gingival samples, whereas AHNAK2 remains a bioinformatic candidate. The resulting nomogram discriminated periodontitis from health in the training dataset. These biomarkers were associated with immune activation and stratified periodontitis tissue samples into three expression-based subgroups. Single-cell analysis highlighted the vascular endothelium, where biomarker expression varied along an inferred pseudotime trajectory, while CellChat predicted altered endothelial–immune communication. The principal value of this signature lies in molecular stratification of periodontitis and in nominating candidates for minimally invasive assays such as gingival crevicular fluid testing. Together, these findings link the amino acid metabolism-related signature to endothelial and immune alterations in periodontitis and define priorities for mechanistic validation.

## Data Availability

The datasets presented in this study can be found in online repositories. The names of the repository/repositories and accession number(s) can be found below: https://www.ncbi.nlm.nih.gov/geo/, GSE16134 https://www.ncbi.nlm.nih.gov/geo/, GSE10334 https://www.ncbi.nlm.nih.gov/geo/, GSE223924 https://www.ncbi.nlm.nih.gov/geo/, GSE164241.
