# Supplementary material for: Integrative bulk and single-cell transcriptomics links amino acid metabolism-related biomarkers to endothelial immune remodeling in periodontitis
Source: Front Immunol. 2026 Oct 28;17:1991235. doi: 10.3389/fimmu.2026.1991235 (PMC13752155; doi:10.3389/fimmu.2026.1991235)
Supplement: Supplementary file 1 [file Table1.docx]

***Supplementary Material***

**1. Supplementary Figures**

**Supplementary Figure1 Identification of TDO2, ICAM2, ACAT2, VWF, FABP4 and AHNAK2 as biomarkers.**

(A) Expression difference analysis of the biomarkers between the PD and CT groups in the validation set GSE10334. ***P < 0.001.

(B) ROC curves of the six candidate biomarkers in the validation dataset GSE10334.

**Supplementary Figure2** **Construction and evaluation of the nomogram model.**

(A) ROC curve of the nomogram model in the GSE10334 (AUC = 0.937). (B) Calibration curve of the nomogram model in the GSE10334. (C) DCA analysis of the nomogram model in the GSE10334. (D) ROC curve of the nomogram model in the GSE223924 (AUC = 0.990). (E) Calibration curve of the nomogram model in the GSE223924. (F) DCA analysis of the nomogram model in the GSE223924.

**Supplementary Figure3 Protein-protein Interaction Network.**

(A) Protein-protein interaction network of the candidate biomarkers and their interacting proteins. (B) Gene-gene association network showing the six candidate biomarkers and their functionally related genes.

**Supplementary Figure4 Immune characteristics of the three periodontitis molecular subtypes.**

(A) MCP-counter-derived abundance scores of 10 immune and stromal cell populations across clusters C1–C3. (B) CIBERSORT-estimated proportions of 22 immune cell types across the three clusters. (C) Expression levels of 28 inflammation-related genes across the three clusters. ns, not significant; *P < 0.05; **P < 0.01; ***P < 0.001.

**Supplementary Figure5 Cell-type distribution and diagnostic biomarker module score.**

(A) Scores of each cell cluster evaluated by the Ro/e system. Ro/e > 1 indicates enrichment, whereas Ro/e < 1 indicates depletion. (B) Diagnostic biomarker module scores across cell types in the CT and PD groups. Scores were calculated from TDO2, ICAM2, ACAT2, VWF, FABP4, and AHNAK2.
