# Supplementary material for: Integrative bulk and single-cell transcriptomics links amino acid metabolism-related biomarkers to endothelial immune remodeling in periodontitis
Source: Front Immunol. 2026 Oct 28;17:1991235. doi: 10.3389/fimmu.2026.1991235 (PMC13752155; doi:10.3389/fimmu.2026.1991235)
Supplement: Supplementary file 2 [file Table3.docx]

**Table 2. Sequences of Primers**

| Name | Sense strand/sense primer (5’-3’) | Antisense strand/antisense primer (5’-3’) |
| --- | --- | --- |
| FABP4 | CAGCACCCTCCTGAAAACTGC | TGGCAAAGCCCACTCCTACT |
| ICAM2 | GCCACAGCCACATTCAACAGCACG | ACAGGGACAGCAACACCGACACCAC |
| VWF | GTGGATGTCACAGCTTGGGC | TCCGAGGTCAAGGTCCCTTCT |
| TDO2 | ACAGAGCACTTCAGGGAGCAT | GGAACCTAGGCTCTTCCCTGT |
| ACAT2 | ATCCTGTGGTCATCGTCTCGG | AGGCACCATTGAAGGAACCTATG |
| GAPDH | AACTCCCATTCTTCCACCTTTGATG | CTGTTGCTGTAGCCATATTCATTGTC |
